# Research on Diagnosis and Management of Postgraduates Mental Health Status Based on BP Neural Network

**DOI:** 10.3389/fpubh.2022.897565

**Published:** 2022-05-10

**Authors:** Jiangning Xie

**Affiliations:** ^1^School of Management, Shandong University, Jinan, China; ^2^Graduate School, Shandong University, Jinan, China

**Keywords:** BP neural network, postgraduate mental health, diagnosis, management, graduate students

## Abstract

As a special group facing the huge pressure of study, employment, life, and other aspects, graduate students have had fierce psychological conflicts. Their mental health status has been greatly affected. Postgraduate mental health problems are related to students' normal life and study and the scientific research work during graduate study. Because of this, this paper uses the BP neural network to establish a diagnostic model for postgraduate mental health status with the dependent variables of SCL-90 psychometric test results to realize the diagnosis of postgraduate' mental health status. The accuracy of the training, validation, and test process of the BP neural network is 98.448, 97.373, 98.128%, and the overall fitting accuracy of the model is 98.273% and the prediction accuracy of graduate mental health is 97.93%. Finally, according to the mental health status of graduate students to ensure the smooth progress of scientific research during graduate students.

## Introduction

The importance of postgraduate mental health is self-evident, whether to the postgraduate or the school, society, and the country. Nowadays, the pace of study, life, and postgraduate scientific research are accelerating. In addition, it is difficult to find a job after graduation, and the psychological pressure of students is increasing rapidly. In many aspects, such as daily study and scientific research, interpersonal communication, emotional life, depression, anxiety, inferiority, and other different psychological problems, which have caused a significant impact on the growth of graduate students. Therefore, attaching great importance to the mental health education of graduate students has become a critical task in graduate student training in various universities.

### Research on the Mental Health of Graduate Students

Since the late 1980s, there have been more and more studies on the mental health problems of college students at home and abroad. However, most of these studies focused on the mental health status and related factors of undergraduate students, while relatively few studies on the quality of mental health in the graduate population. Research on graduate mental health can be roughly divided into two aspects. The first aspect is the causes, performance, and countermeasures of graduate student mental health problems. Ma et al. (1) investigated the mental health status and life pressure of 340 graduate students in a university. The SCL-90 results showed that the detection rate of psychological problems in graduate students was 44.16%, which explains the prevalence of mental health problems in graduate students. Shanmuganandapala (2) also noted that nearly 25 percent of graduate students have mental health problems, such as symptoms of depression, anxiety, or seasonal affective disorders. Posselt (3) constructed a hierarchical clustering model, studied the mental health problems of nearly 20,888 graduate students in 69 universities and compared the prevalence of depression and anxiety in different research fields. The results showed that graduate students majoring in humanities, arts, and architecture have a significantly higher probability of depression and anxiety than graduate students in other majors. On the psychological status of the Chinese Academy of Chinese Medical Sciences, Wang et al. (4) found that the psychological problems of graduate students were coercion (14.02%), interpersonal disorder (7.32%), depression (8.54%), and anxiety (5.49%).

### Research on Management Strategies for Graduate Mental Health Problems

Given the mental health problems of graduate students, many scholars put forward the corresponding management strategies. Among them, Huang et al. (5) discussed the impact of group counseling guided by positive psychology theory on the mental health level of graduate students. The research results show that group counseling based on positive psychology theory can improve the mental health status of graduate students, reduce their negative symptoms and perceptual stress level, and improve their self-acceptance degree. Based on the positive psychological thought, from the perspective of psychological health promotion, Liang et al. (6) constructed a four-level promotion model of graduate mental health that is based on the needs survey of graduate mental health, classification needs as the goal, promotion mode as the core, and effect evaluation as the feedback means. The results showed that good results were achieved.

To sum up, the current graduate mental health problems are mainly from the causes of mental health, the current situation of mental health education, and countermeasures. Most of these studies are theoretical, and few experts quantify the graduate mental health problems and predict their mental health problems through the relevance of the data. Because of this, this paper takes the mental health status of graduate students as the primary research line, obtains the pressure capacity of graduate students in the face of various factors through questionnaire survey, and obtains the mental health evaluation score of graduate students through the SCL-90 questionnaire. Finally, the BP neural network model with the former as the input variable and the latter as the output variable is used to diagnose and manage graduate mental health status.

## Questionnaire and the Model

### Acquisition of Graduate Mental Health Data Based on Questionnaires

#### Questionnaire Design

For the analysis of the factors affecting graduate mental health, this paper from the social, family, school, and individual level summarizes the following 15 influencing factors such as social adaptation, social temptation, employment, future choice, new environment, economy, competition, research, interpersonal communication, scientific research, others, parents expectations, family background, character defects, conflict of interest. According to the 15 factors that affect graduate psychology, the questionnaire is formulated as follows (Table 1 is the main content of the questionnaire):

**Table 1:** Main contents of the questionnaire.

	**Influence factor**	**Importance and self-assessment**					
1	With the rapid development of today's society, the conflict between modern ideas and traditional ideas, and the differences between positive ideological education and popular secular views, make it difficult for you to adapt to society. For the pressure to adapt to society, do you feel?	Score	A	B	C	D	E
		Evaluation	Giant	Big	Normal	Small	Wee
2	In today's social society, harmful social phenomena such as value loss and moral decline, and irrational thoughts and behaviors such as hedonism, money worship, and extreme individualism affect others all the time. Do you feel about the pressure of resisting social temptation?	Score	A	B	C	D	E
		Evaluation	Giant	Big	Normal	Small	Wee
3	With the introduction of competition mechanisms, enterprises have higher and higher requirements for the first degree of graduate students. Facing the pressure of employment, do you feel?	Score	A	B	C	D	E
		Evaluation	Giant	Big	Normal	Small	Wee
4	In the face of the reform of the personnel system of social institutions, society has put forward more stringent employment standards to graduate students. With the expansion of graduate student enrollment, the gold content of master's graduate students has become lower and lower, and the employment pressure is becoming greater and greater. Many students face difficult choices in doctoral study and employment. Do you feel about the pressure on this choice?	Score	A	B	C	D	E
		Evaluation	Giant	Big	Normal	Small	Wee
5	Entering the environment, everything needs to adapt again, leaving their parents to live alone. Some students even leave their city to come to a strange town, facing the pressure caused by such environmental changes, do you feel?	Score	A	B	C	D	E
		Evaluation	Giant	Big	Normal	Small	Wee
6	With the rising prices and the continuous expenditure of various expenses during the graduate years, do you feel about the economic pressure?	Score	A	B	C	D	E
		Evaluation	Giant	Big	Normal	Small	Wee
7	Graduate students are talents selected by the university. They are all talents with excellent academic performance in the class. After coming to graduate school, they come to a new competition stage, and almost everyone has the same starting point of competition. In the face of this competitive pressure, do you feel?	Score	A	B	C	D	E
		Evaluation	Giant	Big	Normal	Small	Wee
8	Facing the triple pressure of graduate scientific research, study, and tutor, do you feel too burdened? Secondly, there is a graduate school, the examination of various qualification certificates, etc. In the face of this learning pressure, do you feel?	Score	A	B	C	D	E
		Evaluation	Giant	Big	Normal	Small	Wee
9	The students come from different regions, with different political and cultural backgrounds and personal habits, especially those in the dormitory. Due to regional differences, there will inevitably be friction in the process of getting along. In the face of these interpersonal pressures, do you feel?	Score	A	B	C	D	E
		Evaluation	Giant	Big	Normal	Small	Wee
10	The postgraduate period is the enlightenment stage of scientific research. Much scientific research work is not conducted during the university. Do you feel about the pressure of facing new knowledge and new fields?	Score	A	B	C	D	E
		Evaluation	Giant	Big	Normal	Small	Wee
11	For students with excellent performance and outstanding performance, it is easy to get various awards from the school, such as scholarships, exceptional student honor, etc., which will inevitably be envied by other students and thoughtfully calculated by others. In the face of these pressures, do you feel?	Score	A	B	C	D	E
		Evaluation	Giant	Big	Normal	Small	Wee
12	For graduate students, they are all the pride of their parents. Many parents let their children prepare to go abroad, study a doctoral study and seek a high salary job in advance. Are they facing the pressure of their parents' strong expectations?	Score	A	B	C	D	E
		Evaluation	Giant	Big	Normal	Small	Wee
13	Each student has a different family background. Those students with ordinary family conditions hope to graduate as soon as possible, work hard, and earn money to support the family. Some families even give up the idea of studying because of financial problems. Do you feel about the pressure of family economic conditions?	Score	A	B	C	D	E
		Evaluation	Giant	Big	Normal	Small	Wee
14	People will inevitably have personality, cowardice, inferiority, withdrawn, narrow, impulsive, irritable, self-centered nature will be reflected in the individual. This kind of personality is often challenging to get along with others. Do you feel about the interpersonal pressure caused by personality defects?	Score	A	B	C	D	E
		Evaluation	Giant	Big	Normal	Small	Wee
15	There is inevitable friction between students, but many students tend to take themselves as the center, think that others have to consider themselves, especially when they cannot meet their expectations in the psychological imbalance, in the face of the pressure caused by this conflict of interest, do you feel?	Score	A	B	C	D	E
		Evaluation	Giant	Big	Normal	Small	Wee

*Data source: Independent writing*.

#### Self-Testing of Graduate Mental Health Based on the Self-Rated Scale of the SCL-90 Test

Symptoms Self-evaluation Scale SCL-90 (7–9) is one of the world's most famous psychological test scales. It is widely used in consultation clinics or hospitals to understand the psychological situation of the patients or patients. It can also evaluate the efficacy of the evolution of the condition before and after consultation. This scale consists of 90 items, including feeling, emotion, thinking, consciousness, behavior, living habits, interpersonal relationship, diet, and sleep, and nine factors are used to reflect nine aspects of psychological symptoms. The S-Assessment Scale SCL-90 judged the mental health status.

### The Neural Network Model

An neural network model consists of some nodes/neurons, set at multiple layers: the input layer, one or more hidden layers and the output layer. Each node/neuron has an activation function, which calculates how much neuron is “stimulated” (10, 11). At each layer, the collections of nodes/neurons transform the input parameters; these parameters are distributed to the next layer, which is described as (1), (2) and (3):


(1) $$\begin{array}{l} z_{j}^{n}=\sum \left(w_{ji}^{\left(1\right)}x^{n-1}i+w_{j0}^{\left(1\right)}\right) \end{array}$$

(2) $$\begin{array}{l} a_{1}^{n}=\sum \left(w_{ij}^{\left(2\right)}z^{n-1}i+w_{10}^{\left(2\right)}\right) \end{array}$$

(3) $$\begin{array}{l} y_{1}^{n}=F\left(a_{1}^{n}\right) \end{array}$$

Where *x* represents the input to the first layer; *z* represents the first layer's output; *i, j* represents the neural network node index; $w_{ji}^{\left(\text{l}\right)}$ represents the weight between the jth node in the *i*th layer and the ith node in the (*i* + 1)th layer; *F(a*${}_{i}^{n}$*)* represents the output value of *i*th node in (*n* + 1)th layer after being activated by the activation function; *w* and *w*_0_ represent the weight and bias between the neurons, which measures the significance of the data passed along the link (synapse). *F(a)* employs the activation function, which employes the hidden layer's aggregated output to calculate output *y*.

The initial weights and biases randomly assigned, the training process continues until the desired output is obtained, which is evaluated by the cost function (4):


(4) $$\begin{array}{l} Ew=\frac{1}{2}\overset{K}{\underset{k=1}{\sum }}y\left(x_{k},w_{k}\right)-t_{k}^{2} \end{array}$$

Where *y* represents the output; *t* represents the desired output, w represent the weight, Levenberg–Marquardt (LM) algorithm is utilized in the neural network training process, which is a variation of gradient descent. The weight and bias of the neural network model are changed during the training process to minimize the error, which is described as function (5):


(5) $$\begin{array}{l} w^{n}=w^{n-1}-{\left(J^{T}J+\mu I\right)}^{-1}Je^{n-1} \end{array}$$

Where *J*=$\frac{\partial E}{\partial w}$represents the full-scale Jacobian matrix related to *w*; *I* represents the identity matrix; *m* represents a combination coefficient; *e* represents the prediction error.

The Levenberg–Marquardt algorithm starts with a forward computation by (1), (2) and (3). The prediction errors of the output layer and the hidden layer are calculated by (6), (7) and (8):


(6) $$\begin{array}{l} e^{\left(3\right)}=y_{1}-t \end{array}$$

(7) $$\begin{array}{l} \delta_{1}^{3}=e_{1}^{3} \end{array}$$

(8) $$\begin{array}{l} \delta_{j}^{2}=w_{1j}\delta_{1}^{3} \end{array}$$

As shown in function (9) and function (10), the Jacobian is calculated by a back-propagation process:


(9) $$\begin{array}{l} \frac{\partial E}{\partial w_{ji}}=\delta_{j}^{\left(2\right)}x_{i} \end{array}$$

(10) $$\begin{array}{l} \frac{\partial E}{\partial w_{1j}}=\delta_{1}^{3}z_{j} \end{array}$$

In the training process of the sample, the learning sample should be processed to make it fluctuate in a certain range. The normalization method is adopted in this paper to process the data to ensure that the data is between 0 and 1, which is written as (11):


(11) $$\begin{array}{l} x_{i}=\frac{{\bar{x}}_{i}-x_{\min}}{x_{\max}-x_{\min}} \end{array}$$

Where ${\bar{x}}_{i}$ represents the average value, *x*_max_ represents the maximum value, *x*_min_ represents the minimum value.

## Results and Discussion

### Questionnaire Results Statistics

The respondents of this questionnaire are 500 graduate students coming from different schools and majors. Five hundred questionnaires were sent out, 487 were recovered, and 461 were valid questionnaires, with an efficiency of 92.2%. For the 15 questions in the questionnaire, options A-E correspond to 1–5 points, respectively, and a higher total score indicates more significant psychological stress. The questions 1–15 and the corresponding SCL90 scores are shown in Table 2 (partial results).

**Table 2:** Questionnaire results.

	**Question**															**SCL-90**
	**1**	**2**	**3**	**4**	**5**	**6**	**7**	**8**	**9**	**10**	**11**	**12**	**13**	**14**	**15**	
1	3	5	5	1	4	4	4	3	5	2	3	1	1	4	3	160
2	2	5	2	5	2	4	1	3	5	1	4	5	1	2	4	163.2
3	1	4	4	1	4	5	2	3	5	4	5	2	1	5	2	156.8
4	5	5	4	5	3	1	1	1	3	1	5	1	1	1	5	137.6
5	2	4	5	5	5	5	4	3	2	5	4	4	2	3	1	179.2
457	1	2	1	1	2	2	1	1	5	3	2	4	3	5	1	121.6
458	4	1	4	1	5	5	1	2	2	3	5	1	3	2	4	147.2
459	1	4	1	2	5	2	3	4	1	2	5	5	5	5	5	176
460	5	2	1	5	5	5	1	5	1	4	3	3	2	4	3	169.6
461	5	1	3	4	4	5	1	2	3	1	1	4	5	4	5	156.8

#### Attention

According to the score standard of SCL90, the total score is more than 160, should be further examination, the standard is more than 200 points that there are obvious psychological problems, can turn to psychological counseling, more than 250 is more serious, need a detailed medical examination, is likely to do targeted psychological treatment or take medicine under the guidance of a doctor.

### Graduate Student Mental Health Diagnosis Model Establishment

Fifteen factors affecting graduate psychology were used as independent variables based on the above questionnaire. The corresponding SCL-90 mental health diagnosis scores were dependent variables to establish the BP neural network model. The data for the training model has randomly selected 400 sets, with 80% training set, 20% test set, and 20% validation set. The remaining 61 sets of data were control groups, and the error between the SCL-90 score and the actual SCL-90 score was analyzed to verify the model's utility (12).

**Figure 1:**
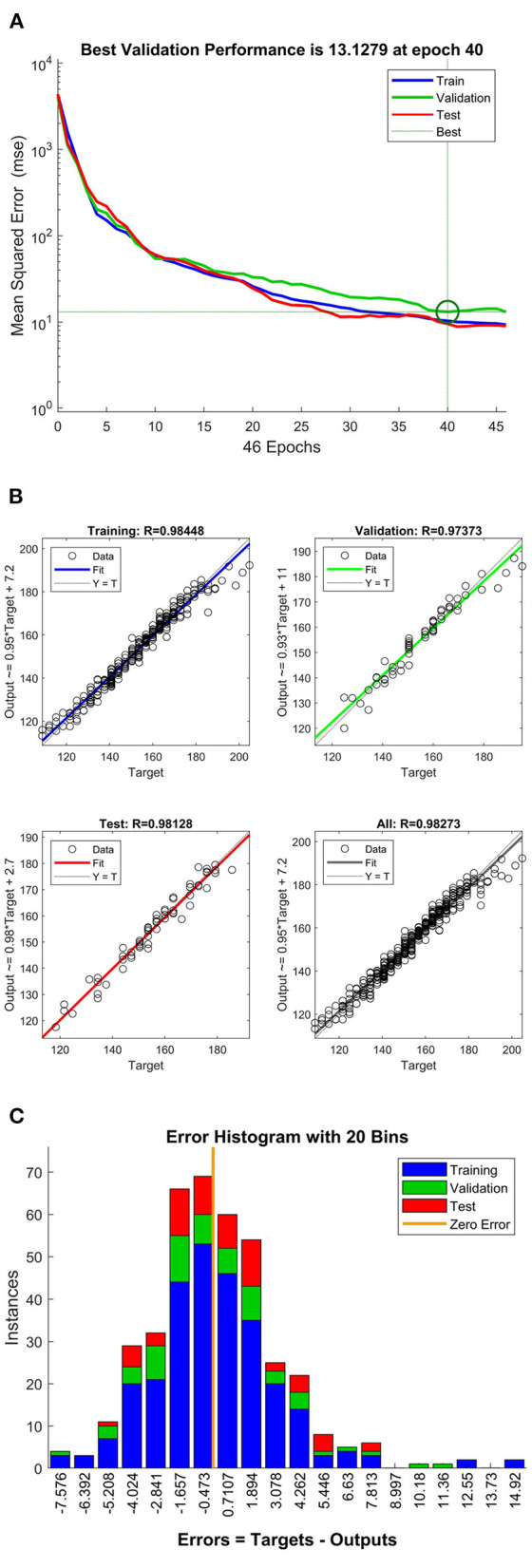
Training process of the model; **(A)** convergence characteristics; **(B)** regression performance of the model; **(C)** error histogram of the model.

The training procedure of this model is shown in Figure 1.

The training process of the BP neural network model is shown in Figure 1A and improves the above neural network configuration. The regression model is initialized in the training process, with the ideal error convergence rate in step 40. Figure 1B shows that high fitting accuracy is obtained after the training process. Then, the accuracy of the training, validation, and test process is 98.448, 97.373, 98.128%, and the overall fitting accuracy of the model is 98.273%. The model's accuracy is high, especially in the test, where the predicted values of the output parameters are close to the reference value. Results show that the relationship between the input and output parameters is modeled correctly. Further prediction/estimation is reliable. Moreover, the error histogram of the model is shown in Figure 1C. For all models, 20 bins are distributed according to Gaussian rules, proving the representativeness of the training data.

### Application of the Graduate Mental Health Diagnostic Model

By establishing the BP neural network model, the mental health of the remaining 61 students was diagnosed, and the questionnaire results of these 61 students were input into the prediction model to obtain the following output results and compare the output results to the actual results of SCL-90, as shown in Table 3.

**Table 3:** Mental health diagnosis results.

	**Actual**	**Predicted**	**Error**		**Actual**	**Predicted**	**Error**		**Actual**	**Predicted**	**Error**
	**value**	**value**	**(%)**		**value**	**value**	**(%)**		**value**	**value**	**(%)**
1	144	147.39	2.35	21	173	171.96	0.60	41	138	134.09	2.83
2	166	169.03	1.83	22	166	169.69	2.22	42	199	189.31	4.87
3	137	139.13	1.55	23	150	148.47	1.02	43	128	134.39	4.99
4	128	127.76	0.19	24	150	152.02	1.35	44	166	156.36	5.81
5	153	156.02	1.97	25	157	158.69	1.08	45	186	183.33	1.44
6	144	143.76	0.17	26	179	178.89	0.06	46	144	142.74	0.87
7	109	116.74	7.10	27	128	129.56	1.22	47	150	149.02	0.65
8	141	141.41	0.29	28	125	125.25	0.20	48	170	173.86	2.27
9	186	180.32	3.05	29	131	131.75	0.57	49	150	146.26	2.49
10	150	152.67	1.78	30	144	143.57	0.30	50	166	173.00	4.22
11	147	148.95	1.33	31	137	132.62	3.20	51	109	114.98	5.49
12	176	180.84	2.75	32	140	137.61	1.71	52	157	160.44	2.19
13	147	145.86	0.78	33	160	156.76	2.03	53	134	131.43	1.92
14	156	149.26	4.32	34	144	141.52	1.72	54	147	152.50	3.74
15	163	164.89	1.16	35	134	136.92	2.18	55	147	144.64	1.61
16	144	142.70	0.90	36	173	176.90	2.25	56	186	181.03	2.67
17	144	145.24	0.86	37	157	160.79	2.41	57	122	126.51	3.70
18	150	153.17	2.11	38	131	127.68	2.53	58	147	147.90	0.61
19	172	168.76	1.88	39	173	178.54	3.20	59	176	179.24	1.84
20	131	129.98	0.78	40	147	144.49	1.71	60	169	166.95	1.21
								61	157	153.39	2.30
Average error (%)	2.07										
Overall accuracy (%)	97.93										

*Data source: Author organizes*.

As shown in Table 2, the trained diagnosis model achieved an excellent diagnostic effect in practical application. The overall accuracy reached 97.93%, an average error of 2.07%. Among them, the largest diagnostic error of 7.10%, through the above analysis, it can be seen that the mental health diagnosis model in practice or has very high practical value, the judgment of students with psychological problems is more accurate, achieved the expected effect.

### Management of Graduate Mental Health Problems

**Figure 2:**
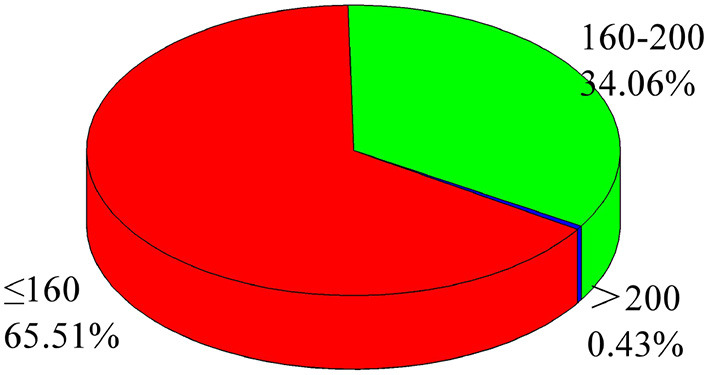
Statistics of mental health scores for graduate students. Data source: Author organizes.

Figure 2 shows the mental health diagnosis scores of the 461 graduate students in this survey. As shown in Figure 2, 34.06% of graduate students had mild psychological problems in the survey results, and 0.43% had obvious psychological problems. Still, they could be solved through psychological counseling. No students in this survey scored more than 250 points, indicating that the surveyed graduate students could solve their psychological problems through positive guidance methods such as psychological counseling.

It can be seen from this survey that the psychological problems of graduate students are common, but they can be solved through positive guidance.

Given the above analysis, this paper proposes the management strategy of graduate psychological problems:

(1) Improve the tutor responsibility system

During graduate school, the relationship between tutor and graduate student is the most important interpersonal relationship. In the training process of graduate students, the communication between tutors and graduate students is the closest. Attention should be paid to building an excellent psychological contract between “mentor and graduate students.” Colleges and universities should improve the tutor responsibility system, give full play to the guiding role of tutors, and clarify the importance of tutors for enhancing the comprehensive ability of graduate students. Graduate tutors should always be strict with themselves in teachers' ethics, set an example in scientific research work, and abide by the principle of integrity.

The tutor has a subtle influence on students 'mental health problems in scientific research and life. The tutor's guidance and evaluation will directly affect the self-efficacy of graduate students. Sometimes the tutor's sentence can greatly encourage students' enthusiasm for scientific research. When students encounter scientific research problems, they should give timely guidance. When students have mastered the experimental techniques and principles in the field, they should lead students to study the significant cutting-edge problems in the area appropriately.

Due to the expansion of graduate enrollment, the number of tutors increases, so it is difficult to consider all graduate students. In order to improve the quality of graduate training, the relevant departments can appropriately control the number of students. According to relevant surveys, some tutors use the “free-range” mode for graduate students and meet less often, so it is difficult to timely understand the students' scientific research status in time, it is difficult for students to get reasonable guidance, and it is easy to cause too much pressure on scientific research. So suggest tutor according to their own and team, appropriate increase for graduate research guidance and humanistic care time, can be through group form, once a week or once every 2 weeks, timely understand everyone's scientific research and life situation, give full play to the mentor lead and research guidance, create a good relationship between teachers and students, reasonable reduce and avoid graduate mental health level, reduce or relieve the pressure of medical graduate research.

Due to the expansion of graduate enrollment, the number of tutors increases, so it is difficult to consider all graduate students. To improve the quality of graduate training, the relevant departments can appropriately control the number of students. According to relevant surveys, some tutors use the “free-range” mode for graduate students and meet less often. So it isn't easy to understand the students' scientific research status in time timely. It is difficult for students to get appropriate guidance, and it is easy to cause too much pressure on scientific research. Therefore, according to their situation and the research group, the tutors can appropriately increase the time of scientific research guidance and humanistic care for graduate students. They can timely understand everyone's scientific research and life situation and give full play to the role of the tutors in the guidance and scientific research guidance. Finally, it will create a good teacher-student relationship, reasonably reduce and avoid the low level of mental health of graduate students, and reduce or relieve the scientific research pressure of medical graduate students.

(2) Strengthen the mental health education in colleges and universities

Graduate mental health education is crucial in the graduate training stage. We should start from the actual situation of mental health education in colleges and universities to strengthen the mental health education of graduate students and effectively help postgraduate students to relieve psychological pressure. Colleges and universities can establish mental health counseling rooms or online consultation topics by psychological doctors. Graduate students can timely solve their problems in life and scientific research and prevent them from deteriorating into psychological issues.

(3) Increase the scientific research support for graduate students

The generation of scientific research results is inseparable from the support of universities for scientific research work. Therefore, to reduce the pressure of students in scientific research, the schools need to provide vital support in the foundation of scientific research. According to the research subject direction of the institute, each department buys perfect public scientific research facilities so that students can obtain scientific research results through experiments and reduce their burden on scientific research.

(4) Counselors actively carry out psychological counseling work

College counselors are a high-quality team with strong combat effectiveness. They should give full play to the role of counselors and strengthen the attention of graduate students. College counselors should pay attention to graduate mental health education. As a healthy vulnerable group, their mental health level is generally low. Female graduate students have delicate and rich feelings, are sensitive to any stimulation, have a changeable mood, have relatively fragile psychology, and are prone to dependence, inferiority, depression, and other psychological problems. Therefore, counselors should pay attention to the mental health of graduate students, especially the mental health of female graduate students, including family situations and love situations. Counselors can set up a mutual aid group for graduate students to fully play the exemplary role of senior teachers and senior brothers.

## Conclusion

Under the background of graduate mental health tension, through the study of the graduate mental health factors, and based on design graduate mental health evaluation questionnaire and SCL-90 mental health evaluation, using the BP neural network model to graduate mental health diagnosis, and verify the feasibility of the diagnostic model. After the final model validation, the prediction accuracy of graduate mental health is 97.93%, which indicates that the mental health diagnostic model established in this paper can evaluate reasonably the mental health status and is feasible.

## Data Availability Statement

The original contributions presented in the study are included in the article/Supplementary Material, further inquiries can be directed to the corresponding author.

## Author Contributions

The author confirms being the sole contributor of this work and has approved it for publication.

## Conflict of Interest

The author declares that the research was conducted in the absence of any commercial or financial relationships that could be construed as a potential conflict of interest.

## Publisher's Note

All claims expressed in this article are solely those of the authors and do not necessarily represent those of their affiliated organizations, or those of the publisher, the editors and the reviewers. Any product that may be evaluated in this article, or claim that may be made by its manufacturer, is not guaranteed or endorsed by the publisher.
