# Supplementary material for: Research on Diagnosis and Management of Postgraduates Mental Health Status Based on BP Neural Network
Source: Front Public Health. 2022 May 10;10:897565. doi: 10.3389/fpubh.2022.897565 (PMC9128816; doi:10.3389/fpubh.2022.897565)
Supplement: Supplementary file 1 [file Table_1.DOCX]

**Appendix: Questionnaire data of 461 graduate students (raw data of model training)**

|  | 1 | 2 | 3 | 4 | 5 | 6 | 7 | 8 | 9 | 10 | 11 | 12 | 13 | 14 | 15 | SCL-90 |
| --- | --- | --- | --- | --- | --- | --- | --- | --- | --- | --- | --- | --- | --- | --- | --- | --- |
| 1 | 3 | 5 | 5 | 1 | 4 | 4 | 4 | 3 | 5 | 2 | 3 | 1 | 1 | 4 | 3 | 160 |
| 2 | 2 | 5 | 2 | 5 | 2 | 4 | 1 | 3 | 5 | 1 | 4 | 5 | 1 | 2 | 4 | 163 |
| 3 | 1 | 4 | 4 | 1 | 4 | 5 | 2 | 3 | 5 | 4 | 5 | 2 | 1 | 5 | 2 | 157 |
| 4 | 5 | 5 | 4 | 5 | 3 | 1 | 1 | 1 | 3 | 1 | 5 | 1 | 1 | 1 | 5 | 138 |
| 5 | 2 | 4 | 5 | 5 | 5 | 5 | 4 | 3 | 2 | 5 | 4 | 4 | 2 | 3 | 1 | 179 |
| 6 | 1 | 1 | 3 | 1 | 5 | 5 | 5 | 5 | 5 | 2 | 4 | 2 | 2 | 1 | 4 | 154 |
| 7 | 2 | 3 | 2 | 3 | 1 | 3 | 2 | 1 | 4 | 5 | 1 | 1 | 5 | 2 | 4 | 131 |
| 8 | 3 | 2 | 4 | 3 | 5 | 2 | 2 | 4 | 4 | 1 | 3 | 2 | 5 | 4 | 3 | 157 |
| 9 | 1 | 4 | 4 | 1 | 2 | 5 | 3 | 5 | 4 | 4 | 1 | 4 | 5 | 5 | 4 | 176 |
| 10 | 5 | 4 | 3 | 4 | 2 | 4 | 1 | 5 | 5 | 1 | 2 | 2 | 2 | 4 | 2 | 163 |
| 11 | 1 | 2 | 3 | 1 | 3 | 1 | 2 | 1 | 5 | 4 | 4 | 1 | 4 | 3 | 5 | 131 |
| 12 | 4 | 2 | 5 | 2 | 4 | 1 | 3 | 1 | 4 | 1 | 4 | 5 | 2 | 1 | 2 | 144 |
| 13 | 2 | 2 | 1 | 4 | 2 | 5 | 5 | 1 | 5 | 5 | 5 | 1 | 3 | 3 | 3 | 166 |
| 14 | 1 | 4 | 3 | 1 | 4 | 1 | 3 | 2 | 1 | 2 | 2 | 2 | 5 | 4 | 1 | 125 |
| 15 | 5 | 3 | 4 | 3 | 1 | 1 | 4 | 2 | 5 | 5 | 2 | 2 | 1 | 1 | 2 | 141 |
| 16 | 1 | 5 | 1 | 3 | 1 | 1 | 4 | 5 | 1 | 5 | 2 | 4 | 3 | 3 | 4 | 147 |
| 17 | 4 | 4 | 5 | 3 | 5 | 2 | 1 | 4 | 1 | 4 | 3 | 5 | 1 | 4 | 2 | 160 |
| 18 | 2 | 2 | 5 | 4 | 2 | 5 | 4 | 1 | 1 | 2 | 1 | 4 | 1 | 4 | 2 | 131 |
| 19 | 3 | 2 | 4 | 3 | 3 | 2 | 1 | 4 | 3 | 4 | 1 | 1 | 3 | 4 | 5 | 141 |
| 20 | 1 | 2 | 3 | 2 | 4 | 3 | 5 | 1 | 1 | 4 | 2 | 2 | 4 | 4 | 2 | 131 |
| 21 | 3 | 5 | 1 | 4 | 1 | 5 | 4 | 5 | 5 | 2 | 3 | 3 | 3 | 5 | 4 | 186 |
| 22 | 4 | 5 | 3 | 4 | 3 | 2 | 4 | 5 | 3 | 1 | 3 | 4 | 4 | 2 | 4 | 179 |
| 23 | 3 | 4 | 1 | 4 | 4 | 1 | 5 | 3 | 3 | 5 | 2 | 4 | 4 | 3 | 3 | 173 |
| 24 | 5 | 1 | 1 | 3 | 5 | 2 | 1 | 5 | 3 | 2 | 4 | 4 | 3 | 3 | 4 | 160 |
| 25 | 3 | 4 | 5 | 2 | 3 | 4 | 3 | 3 | 2 | 5 | 5 | 5 | 2 | 2 | 5 | 176 |
| 26 | 2 | 4 | 4 | 5 | 3 | 2 | 2 | 4 | 2 | 3 | 2 | 3 | 2 | 3 | 5 | 157 |
| 27 | 5 | 2 | 3 | 1 | 3 | 5 | 5 | 1 | 2 | 2 | 5 | 1 | 5 | 4 | 4 | 163 |
| 28 | 2 | 2 | 3 | 3 | 1 | 5 | 3 | 2 | 4 | 5 | 1 | 1 | 1 | 2 | 5 | 134 |
| 29 | 1 | 4 | 2 | 2 | 3 | 5 | 3 | 2 | 5 | 1 | 3 | 4 | 1 | 2 | 2 | 138 |
| 30 | 5 | 2 | 1 | 1 | 4 | 2 | 4 | 4 | 5 | 4 | 5 | 1 | 5 | 5 | 5 | 186 |
| 31 | 3 | 5 | 4 | 4 | 1 | 1 | 4 | 3 | 1 | 4 | 1 | 1 | 1 | 2 | 3 | 134 |
| 32 | 4 | 2 | 3 | 2 | 2 | 5 | 4 | 3 | 4 | 3 | 2 | 4 | 2 | 3 | 4 | 163 |
| 33 | 2 | 4 | 1 | 3 | 3 | 4 | 4 | 2 | 5 | 4 | 1 | 5 | 3 | 2 | 4 | 166 |
| 34 | 1 | 1 | 2 | 5 | 2 | 3 | 1 | 4 | 3 | 1 | 2 | 5 | 4 | 3 | 3 | 134 |
| 35 | 5 | 2 | 4 | 3 | 2 | 1 | 5 | 1 | 2 | 2 | 4 | 3 | 2 | 5 | 5 | 163 |
| 36 | 2 | 5 | 4 | 4 | 1 | 4 | 3 | 1 | 5 | 1 | 5 | 2 | 5 | 3 | 1 | 160 |
| 37 | 2 | 5 | 4 | 5 | 5 | 4 | 4 | 2 | 4 | 4 | 2 | 5 | 5 | 3 | 3 | 195 |
| 38 | 2 | 3 | 2 | 5 | 3 | 4 | 3 | 2 | 2 | 3 | 2 | 5 | 3 | 4 | 1 | 144 |
| 39 | 3 | 2 | 2 | 3 | 3 | 5 | 1 | 2 | 5 | 2 | 5 | 5 | 4 | 3 | 1 | 163 |
| 40 | 2 | 3 | 5 | 1 | 1 | 5 | 3 | 4 | 1 | 2 | 4 | 1 | 2 | 1 | 5 | 134 |
| 41 | 3 | 3 | 4 | 5 | 1 | 3 | 4 | 4 | 5 | 1 | 1 | 5 | 5 | 4 | 1 | 160 |
| 42 | 2 | 4 | 1 | 4 | 4 | 3 | 3 | 4 | 3 | 4 | 4 | 3 | 3 | 4 | 3 | 163 |
| 43 | 4 | 5 | 3 | 3 | 1 | 3 | 3 | 1 | 2 | 2 | 3 | 1 | 3 | 5 | 3 | 144 |
| 44 | 1 | 2 | 1 | 2 | 2 | 2 | 4 | 5 | 2 | 3 | 1 | 2 | 3 | 2 | 4 | 131 |
| 45 | 1 | 2 | 2 | 1 | 5 | 5 | 4 | 1 | 1 | 5 | 4 | 3 | 3 | 5 | 1 | 147 |
| 46 | 4 | 2 | 1 | 1 | 5 | 1 | 3 | 1 | 3 | 3 | 3 | 3 | 3 | 5 | 4 | 141 |
| 47 | 1 | 4 | 2 | 1 | 3 | 1 | 2 | 1 | 3 | 2 | 2 | 5 | 1 | 2 | 4 | 122 |
| 48 | 4 | 4 | 2 | 5 | 5 | 2 | 3 | 4 | 4 | 5 | 4 | 3 | 1 | 1 | 5 | 170 |
| 49 | 3 | 3 | 5 | 3 | 3 | 1 | 4 | 2 | 2 | 3 | 3 | 5 | 5 | 5 | 5 | 173 |
| 50 | 1 | 4 | 3 | 5 | 5 | 1 | 5 | 5 | 5 | 4 | 2 | 2 | 5 | 5 | 3 | 192 |
| 51 | 2 | 5 | 2 | 5 | 1 | 5 | 3 | 2 | 5 | 1 | 2 | 1 | 5 | 1 | 5 | 157 |
| 52 | 5 | 4 | 4 | 4 | 5 | 1 | 4 | 2 | 1 | 2 | 1 | 2 | 5 | 1 | 4 | 154 |
| 53 | 4 | 5 | 5 | 5 | 2 | 5 | 2 | 3 | 2 | 2 | 1 | 1 | 3 | 3 | 3 | 154 |
| 54 | 3 | 1 | 4 | 4 | 3 | 4 | 1 | 1 | 1 | 4 | 4 | 5 | 5 | 4 | 2 | 150 |
| 55 | 2 | 4 | 4 | 2 | 3 | 5 | 1 | 3 | 1 | 4 | 5 | 2 | 1 | 1 | 2 | 144 |
| 56 | 1 | 1 | 2 | 4 | 1 | 4 | 3 | 1 | 1 | 3 | 3 | 4 | 2 | 2 | 5 | 128 |
| 57 | 4 | 3 | 3 | 1 | 3 | 3 | 5 | 4 | 5 | 3 | 1 | 4 | 4 | 2 | 5 | 176 |
| 58 | 4 | 5 | 1 | 2 | 4 | 2 | 1 | 5 | 3 | 5 | 2 | 4 | 3 | 1 | 4 | 160 |
| 59 | 4 | 2 | 2 | 5 | 1 | 1 | 5 | 1 | 4 | 2 | 1 | 4 | 4 | 3 | 5 | 150 |
| 60 | 1 | 2 | 3 | 1 | 3 | 1 | 4 | 3 | 1 | 2 | 3 | 4 | 2 | 4 | 5 | 141 |
| 61 | 3 | 3 | 5 | 5 | 2 | 2 | 2 | 4 | 3 | 5 | 1 | 3 | 4 | 3 | 1 | 154 |
| 62 | 1 | 1 | 1 | 4 | 2 | 3 | 4 | 4 | 4 | 1 | 2 | 1 | 2 | 4 | 1 | 115 |
| 63 | 2 | 5 | 1 | 2 | 1 | 2 | 5 | 2 | 1 | 2 | 5 | 1 | 3 | 1 | 1 | 112 |
| 64 | 3 | 4 | 4 | 4 | 1 | 3 | 5 | 2 | 4 | 3 | 5 | 3 | 3 | 4 | 4 | 182 |
| 65 | 5 | 2 | 5 | 1 | 3 | 3 | 5 | 3 | 4 | 1 | 1 | 1 | 4 | 4 | 3 | 154 |
| 66 | 3 | 4 | 3 | 3 | 1 | 5 | 5 | 5 | 2 | 4 | 5 | 5 | 1 | 5 | 5 | 186 |
| 67 | 3 | 2 | 1 | 5 | 4 | 1 | 2 | 1 | 4 | 3 | 3 | 2 | 1 | 3 | 4 | 138 |
| 68 | 4 | 2 | 1 | 3 | 5 | 3 | 5 | 1 | 1 | 5 | 5 | 3 | 4 | 1 | 1 | 144 |
| 69 | 4 | 5 | 5 | 5 | 2 | 2 | 4 | 1 | 2 | 3 | 5 | 5 | 2 | 5 | 2 | 173 |
| 70 | 3 | 2 | 4 | 1 | 5 | 1 | 1 | 1 | 3 | 3 | 4 | 2 | 5 | 5 | 1 | 141 |
| 71 | 4 | 4 | 3 | 2 | 1 | 5 | 3 | 4 | 1 | 4 | 3 | 1 | 4 | 4 | 3 | 163 |
| 72 | 5 | 3 | 2 | 4 | 2 | 5 | 4 | 5 | 4 | 1 | 1 | 3 | 3 | 4 | 1 | 157 |
| 73 | 2 | 2 | 3 | 2 | 3 | 2 | 2 | 5 | 3 | 2 | 5 | 4 | 2 | 4 | 5 | 160 |
| 74 | 4 | 4 | 4 | 1 | 5 | 4 | 1 | 5 | 2 | 3 | 2 | 2 | 5 | 5 | 1 | 163 |
| 75 | 4 | 1 | 5 | 5 | 3 | 1 | 4 | 1 | 1 | 3 | 5 | 2 | 1 | 1 | 4 | 141 |
| 76 | 5 | 2 | 4 | 2 | 1 | 4 | 5 | 5 | 4 | 5 | 2 | 2 | 2 | 1 | 2 | 163 |
| 77 | 2 | 2 | 5 | 3 | 5 | 5 | 4 | 4 | 4 | 3 | 5 | 4 | 4 | 4 | 4 | 202 |
| 78 | 3 | 5 | 1 | 1 | 4 | 3 | 1 | 5 | 4 | 5 | 3 | 2 | 1 | 5 | 3 | 157 |
| 79 | 4 | 5 | 5 | 4 | 5 | 3 | 1 | 3 | 4 | 3 | 5 | 1 | 5 | 1 | 5 | 176 |
| 80 | 3 | 4 | 4 | 3 | 2 | 3 | 5 | 4 | 5 | 3 | 2 | 1 | 4 | 5 | 2 | 173 |
| 81 | 2 | 2 | 1 | 5 | 4 | 3 | 1 | 5 | 1 | 4 | 3 | 5 | 1 | 5 | 2 | 154 |
| 82 | 3 | 3 | 4 | 4 | 1 | 4 | 4 | 4 | 5 | 2 | 5 | 2 | 4 | 4 | 3 | 173 |
| 83 | 1 | 4 | 1 | 3 | 2 | 1 | 2 | 1 | 4 | 5 | 1 | 2 | 1 | 4 | 1 | 118 |
| 84 | 2 | 1 | 4 | 4 | 5 | 5 | 1 | 1 | 1 | 5 | 4 | 3 | 4 | 5 | 2 | 157 |
| 85 | 4 | 2 | 3 | 2 | 3 | 4 | 4 | 5 | 3 | 4 | 3 | 1 | 2 | 5 | 5 | 163 |
| 86 | 1 | 3 | 2 | 1 | 2 | 1 | 1 | 2 | 3 | 2 | 3 | 4 | 2 | 3 | 3 | 109 |
| 87 | 2 | 1 | 4 | 4 | 1 | 5 | 2 | 2 | 4 | 2 | 1 | 2 | 3 | 1 | 5 | 128 |
| 88 | 3 | 4 | 5 | 2 | 1 | 2 | 3 | 4 | 5 | 5 | 4 | 5 | 4 | 2 | 2 | 176 |
| 89 | 4 | 1 | 2 | 2 | 1 | 5 | 3 | 3 | 2 | 2 | 2 | 3 | 1 | 4 | 1 | 122 |
| 90 | 3 | 3 | 2 | 3 | 5 | 1 | 5 | 4 | 5 | 3 | 4 | 1 | 4 | 1 | 4 | 163 |
| 91 | 1 | 1 | 5 | 5 | 3 | 4 | 4 | 1 | 2 | 2 | 3 | 4 | 3 | 2 | 5 | 150 |
| 92 | 2 | 3 | 4 | 2 | 3 | 2 | 5 | 2 | 1 | 4 | 5 | 1 | 3 | 4 | 2 | 147 |
| 93 | 4 | 3 | 3 | 5 | 5 | 3 | 1 | 4 | 3 | 4 | 5 | 3 | 4 | 3 | 1 | 179 |
| 94 | 4 | 3 | 2 | 3 | 2 | 5 | 4 | 5 | 1 | 3 | 3 | 1 | 4 | 2 | 4 | 157 |
| 95 | 4 | 5 | 1 | 2 | 3 | 5 | 5 | 5 | 2 | 3 | 3 | 3 | 5 | 2 | 2 | 170 |
| 96 | 1 | 5 | 3 | 4 | 5 | 5 | 1 | 4 | 1 | 1 | 3 | 4 | 4 | 4 | 1 | 154 |
| 97 | 1 | 4 | 1 | 1 | 1 | 2 | 5 | 1 | 3 | 5 | 1 | 5 | 2 | 4 | 3 | 141 |
| 98 | 4 | 4 | 1 | 3 | 3 | 2 | 4 | 2 | 1 | 2 | 4 | 2 | 4 | 5 | 5 | 163 |
| 99 | 2 | 1 | 5 | 4 | 4 | 3 | 5 | 5 | 1 | 4 | 1 | 3 | 2 | 3 | 3 | 150 |
| 100 | 5 | 5 | 1 | 1 | 4 | 1 | 1 | 5 | 5 | 5 | 3 | 2 | 4 | 5 | 3 | 173 |
| 101 | 2 | 5 | 3 | 2 | 2 | 2 | 1 | 2 | 3 | 4 | 2 | 5 | 3 | 1 | 1 | 138 |
| 102 | 1 | 5 | 5 | 4 | 3 | 5 | 3 | 4 | 4 | 4 | 1 | 3 | 5 | 2 | 3 | 179 |
| 103 | 3 | 1 | 3 | 3 | 2 | 2 | 4 | 3 | 1 | 1 | 5 | 3 | 2 | 4 | 4 | 144 |
| 104 | 5 | 3 | 4 | 3 | 5 | 2 | 2 | 2 | 4 | 5 | 2 | 4 | 3 | 4 | 1 | 170 |
| 105 | 3 | 3 | 4 | 3 | 4 | 1 | 5 | 1 | 1 | 5 | 3 | 5 | 4 | 1 | 2 | 157 |
| 106 | 1 | 2 | 2 | 3 | 3 | 5 | 1 | 5 | 3 | 2 | 5 | 1 | 3 | 3 | 1 | 131 |
| 107 | 2 | 3 | 2 | 4 | 3 | 1 | 1 | 1 | 3 | 5 | 1 | 2 | 4 | 3 | 2 | 134 |
| 108 | 4 | 5 | 4 | 1 | 2 | 1 | 4 | 2 | 5 | 1 | 4 | 3 | 4 | 2 | 3 | 150 |
| 109 | 4 | 2 | 1 | 3 | 5 | 5 | 5 | 3 | 1 | 2 | 5 | 2 | 4 | 3 | 3 | 170 |
| 110 | 1 | 3 | 5 | 4 | 5 | 3 | 5 | 3 | 2 | 2 | 2 | 2 | 1 | 2 | 1 | 141 |
| 111 | 4 | 4 | 5 | 4 | 3 | 1 | 1 | 4 | 3 | 3 | 1 | 1 | 1 | 3 | 2 | 141 |
| 112 | 5 | 4 | 4 | 5 | 2 | 3 | 5 | 4 | 1 | 2 | 3 | 3 | 2 | 4 | 2 | 166 |
| 113 | 5 | 4 | 1 | 5 | 1 | 2 | 1 | 5 | 4 | 3 | 3 | 5 | 5 | 2 | 3 | 166 |
| 114 | 4 | 3 | 1 | 2 | 3 | 1 | 3 | 3 | 2 | 1 | 3 | 1 | 4 | 4 | 5 | 144 |
| 115 | 3 | 4 | 1 | 5 | 5 | 2 | 1 | 5 | 2 | 3 | 2 | 5 | 4 | 4 | 5 | 170 |
| 116 | 3 | 4 | 3 | 1 | 4 | 3 | 2 | 5 | 4 | 2 | 4 | 1 | 3 | 3 | 4 | 160 |
| 117 | 5 | 5 | 2 | 2 | 5 | 3 | 5 | 3 | 4 | 1 | 4 | 2 | 2 | 5 | 3 | 173 |
| 118 | 5 | 2 | 1 | 2 | 5 | 5 | 3 | 1 | 5 | 2 | 1 | 2 | 3 | 4 | 2 | 147 |
| 119 | 4 | 1 | 3 | 1 | 2 | 2 | 5 | 5 | 3 | 5 | 5 | 1 | 1 | 5 | 5 | 163 |
| 120 | 3 | 2 | 1 | 5 | 4 | 1 | 3 | 4 | 2 | 4 | 3 | 2 | 5 | 3 | 3 | 160 |
| 121 | 3 | 5 | 2 | 4 | 3 | 1 | 1 | 3 | 5 | 1 | 4 | 3 | 1 | 4 | 2 | 141 |
| 122 | 5 | 1 | 5 | 4 | 2 | 2 | 1 | 5 | 3 | 5 | 3 | 1 | 4 | 4 | 1 | 163 |
| 123 | 3 | 1 | 4 | 2 | 5 | 2 | 2 | 2 | 4 | 3 | 2 | 3 | 5 | 1 | 1 | 138 |
| 124 | 3 | 3 | 3 | 1 | 3 | 2 | 2 | 3 | 4 | 4 | 2 | 1 | 4 | 2 | 2 | 128 |
| 125 | 3 | 1 | 3 | 1 | 3 | 5 | 1 | 1 | 4 | 5 | 3 | 5 | 4 | 4 | 3 | 150 |
| 126 | 5 | 1 | 4 | 5 | 2 | 2 | 1 | 3 | 2 | 5 | 2 | 3 | 4 | 3 | 3 | 160 |
| 127 | 2 | 1 | 5 | 4 | 2 | 5 | 5 | 1 | 2 | 1 | 2 | 1 | 3 | 1 | 2 | 131 |
| 128 | 4 | 3 | 1 | 2 | 5 | 1 | 1 | 1 | 4 | 2 | 2 | 2 | 3 | 2 | 1 | 125 |
| 129 | 2 | 2 | 3 | 3 | 3 | 2 | 2 | 5 | 5 | 2 | 3 | 5 | 1 | 4 | 1 | 144 |
| 130 | 2 | 3 | 5 | 3 | 3 | 4 | 2 | 5 | 3 | 3 | 4 | 2 | 5 | 2 | 2 | 157 |
| 131 | 5 | 3 | 5 | 1 | 1 | 5 | 3 | 3 | 4 | 5 | 2 | 4 | 1 | 3 | 4 | 170 |
| 132 | 2 | 5 | 5 | 2 | 4 | 2 | 2 | 4 | 2 | 3 | 5 | 2 | 4 | 2 | 4 | 163 |
| 133 | 4 | 3 | 5 | 4 | 3 | 2 | 4 | 3 | 1 | 1 | 5 | 3 | 5 | 1 | 2 | 154 |
| 134 | 5 | 3 | 2 | 4 | 1 | 3 | 1 | 3 | 2 | 4 | 5 | 5 | 1 | 4 | 2 | 160 |
| 135 | 5 | 3 | 1 | 5 | 5 | 5 | 5 | 1 | 4 | 5 | 2 | 5 | 5 | 1 | 1 | 176 |
| 136 | 5 | 4 | 4 | 1 | 5 | 4 | 5 | 1 | 5 | 2 | 2 | 4 | 5 | 5 | 3 | 179 |
| 137 | 4 | 5 | 2 | 2 | 5 | 5 | 3 | 3 | 2 | 3 | 3 | 1 | 5 | 3 | 2 | 157 |
| 138 | 5 | 2 | 1 | 2 | 4 | 3 | 1 | 4 | 3 | 2 | 3 | 4 | 2 | 5 | 1 | 141 |
| 139 | 1 | 2 | 3 | 3 | 1 | 3 | 5 | 5 | 4 | 5 | 5 | 2 | 4 | 4 | 4 | 179 |
| 140 | 5 | 2 | 5 | 4 | 4 | 4 | 3 | 1 | 5 | 2 | 1 | 2 | 1 | 5 | 3 | 157 |
| 141 | 3 | 4 | 5 | 1 | 1 | 5 | 2 | 5 | 5 | 5 | 1 | 1 | 4 | 4 | 3 | 160 |
| 142 | 5 | 3 | 1 | 4 | 4 | 3 | 3 | 5 | 2 | 2 | 3 | 4 | 4 | 5 | 1 | 163 |
| 143 | 4 | 2 | 3 | 5 | 2 | 1 | 5 | 3 | 1 | 5 | 3 | 1 | 3 | 5 | 1 | 154 |
| 144 | 3 | 2 | 2 | 1 | 2 | 2 | 3 | 4 | 1 | 3 | 3 | 5 | 4 | 1 | 5 | 134 |
| 145 | 5 | 5 | 5 | 3 | 4 | 4 | 1 | 1 | 4 | 3 | 1 | 4 | 3 | 1 | 3 | 166 |
| 146 | 1 | 5 | 5 | 5 | 4 | 3 | 2 | 3 | 1 | 2 | 1 | 1 | 3 | 4 | 2 | 150 |
| 147 | 1 | 3 | 4 | 5 | 1 | 4 | 5 | 4 | 1 | 5 | 3 | 1 | 3 | 2 | 4 | 150 |
| 148 | 3 | 3 | 5 | 5 | 1 | 2 | 2 | 5 | 2 | 3 | 4 | 4 | 5 | 5 | 5 | 182 |
| 149 | 4 | 5 | 2 | 1 | 3 | 5 | 5 | 4 | 4 | 1 | 5 | 4 | 5 | 4 | 5 | 189 |
| 150 | 4 | 3 | 2 | 3 | 5 | 2 | 2 | 2 | 4 | 2 | 1 | 2 | 2 | 5 | 5 | 147 |
| 151 | 2 | 1 | 1 | 4 | 4 | 3 | 2 | 1 | 5 | 3 | 3 | 1 | 3 | 1 | 4 | 125 |
| 152 | 1 | 3 | 1 | 5 | 5 | 1 | 4 | 3 | 2 | 3 | 4 | 3 | 2 | 2 | 1 | 141 |
| 153 | 2 | 2 | 4 | 5 | 3 | 4 | 1 | 2 | 4 | 5 | 5 | 3 | 3 | 2 | 1 | 150 |
| 154 | 4 | 2 | 3 | 5 | 5 | 2 | 4 | 3 | 2 | 2 | 3 | 3 | 4 | 3 | 1 | 157 |
| 155 | 3 | 3 | 3 | 2 | 2 | 5 | 3 | 3 | 5 | 3 | 1 | 5 | 4 | 2 | 1 | 154 |
| 156 | 4 | 2 | 3 | 5 | 2 | 3 | 2 | 1 | 4 | 4 | 5 | 3 | 5 | 3 | 2 | 160 |
| 157 | 5 | 2 | 1 | 4 | 5 | 3 | 3 | 4 | 3 | 2 | 3 | 4 | 3 | 3 | 5 | 163 |
| 158 | 2 | 3 | 4 | 4 | 3 | 5 | 2 | 3 | 5 | 4 | 2 | 2 | 1 | 4 | 3 | 163 |
| 159 | 1 | 2 | 2 | 2 | 1 | 5 | 1 | 3 | 5 | 5 | 4 | 5 | 1 | 4 | 3 | 157 |
| 160 | 2 | 4 | 2 | 1 | 1 | 4 | 4 | 4 | 4 | 5 | 2 | 2 | 4 | 4 | 1 | 144 |
| 161 | 4 | 5 | 4 | 1 | 2 | 2 | 4 | 3 | 1 | 3 | 1 | 4 | 1 | 5 | 4 | 150 |
| 162 | 2 | 3 | 5 | 3 | 1 | 4 | 1 | 3 | 4 | 1 | 2 | 5 | 5 | 4 | 5 | 160 |
| 163 | 2 | 3 | 1 | 5 | 2 | 5 | 4 | 5 | 1 | 2 | 2 | 3 | 1 | 5 | 3 | 157 |
| 164 | 4 | 3 | 2 | 2 | 3 | 3 | 3 | 5 | 4 | 1 | 1 | 2 | 2 | 2 | 2 | 141 |
| 165 | 2 | 4 | 1 | 1 | 2 | 3 | 5 | 4 | 2 | 2 | 5 | 2 | 5 | 3 | 1 | 150 |
| 166 | 1 | 1 | 1 | 1 | 5 | 1 | 2 | 5 | 3 | 4 | 2 | 5 | 4 | 3 | 1 | 128 |
| 167 | 2 | 5 | 5 | 4 | 3 | 3 | 3 | 3 | 2 | 1 | 4 | 3 | 4 | 5 | 3 | 166 |
| 168 | 5 | 1 | 2 | 1 | 4 | 2 | 5 | 1 | 2 | 4 | 4 | 1 | 3 | 5 | 5 | 157 |
| 169 | 3 | 4 | 3 | 4 | 2 | 1 | 2 | 3 | 5 | 1 | 4 | 1 | 4 | 1 | 5 | 150 |
| 170 | 2 | 4 | 5 | 4 | 1 | 2 | 2 | 4 | 1 | 3 | 5 | 3 | 1 | 5 | 1 | 150 |
| 171 | 2 | 1 | 5 | 4 | 5 | 2 | 5 | 2 | 2 | 2 | 4 | 5 | 2 | 5 | 3 | 163 |
| 172 | 3 | 1 | 5 | 3 | 5 | 4 | 2 | 4 | 3 | 3 | 3 | 2 | 5 | 2 | 3 | 160 |
| 173 | 2 | 1 | 5 | 3 | 3 | 4 | 1 | 1 | 4 | 1 | 3 | 1 | 3 | 2 | 1 | 125 |
| 174 | 4 | 5 | 2 | 2 | 2 | 1 | 3 | 3 | 4 | 4 | 4 | 2 | 3 | 2 | 5 | 163 |
| 175 | 3 | 2 | 3 | 1 | 3 | 3 | 3 | 4 | 2 | 5 | 4 | 5 | 2 | 4 | 4 | 157 |
| 176 | 5 | 2 | 3 | 5 | 4 | 3 | 1 | 2 | 3 | 2 | 5 | 5 | 3 | 5 | 5 | 186 |
| 177 | 3 | 3 | 2 | 4 | 3 | 3 | 4 | 4 | 5 | 4 | 3 | 2 | 1 | 5 | 3 | 160 |
| 178 | 5 | 4 | 5 | 2 | 4 | 2 | 5 | 5 | 1 | 1 | 4 | 4 | 2 | 3 | 2 | 166 |
| 179 | 1 | 2 | 3 | 1 | 2 | 2 | 3 | 4 | 2 | 1 | 5 | 5 | 3 | 4 | 4 | 141 |
| 180 | 1 | 5 | 5 | 4 | 1 | 2 | 5 | 5 | 1 | 5 | 5 | 2 | 3 | 2 | 4 | 170 |
| 181 | 1 | 1 | 3 | 3 | 1 | 1 | 5 | 3 | 3 | 5 | 4 | 5 | 3 | 1 | 5 | 144 |
| 182 | 3 | 3 | 4 | 5 | 3 | 1 | 3 | 3 | 2 | 5 | 1 | 2 | 3 | 1 | 3 | 147 |
| 183 | 5 | 2 | 2 | 4 | 1 | 4 | 4 | 5 | 1 | 3 | 4 | 5 | 4 | 3 | 5 | 173 |
| 184 | 4 | 1 | 5 | 2 | 3 | 5 | 4 | 1 | 3 | 4 | 2 | 2 | 3 | 3 | 1 | 141 |
| 185 | 4 | 1 | 4 | 2 | 2 | 1 | 5 | 3 | 3 | 3 | 2 | 5 | 4 | 5 | 4 | 160 |
| 186 | 5 | 4 | 2 | 1 | 5 | 3 | 3 | 2 | 3 | 1 | 5 | 2 | 2 | 5 | 4 | 166 |
| 187 | 2 | 5 | 3 | 1 | 5 | 2 | 2 | 3 | 2 | 2 | 5 | 4 | 3 | 5 | 1 | 147 |
| 188 | 5 | 3 | 2 | 4 | 4 | 5 | 5 | 2 | 5 | 3 | 5 | 3 | 4 | 1 | 1 | 170 |
| 189 | 4 | 2 | 5 | 5 | 1 | 2 | 2 | 5 | 5 | 3 | 5 | 3 | 5 | 2 | 3 | 182 |
| 190 | 4 | 1 | 2 | 2 | 1 | 5 | 1 | 4 | 1 | 1 | 1 | 4 | 2 | 2 | 2 | 112 |
| 191 | 3 | 2 | 2 | 5 | 3 | 2 | 5 | 3 | 4 | 2 | 5 | 2 | 5 | 2 | 2 | 154 |
| 192 | 3 | 2 | 2 | 2 | 2 | 3 | 3 | 1 | 3 | 2 | 5 | 3 | 4 | 1 | 4 | 141 |
| 193 | 1 | 3 | 1 | 5 | 1 | 1 | 2 | 4 | 4 | 1 | 2 | 5 | 3 | 5 | 5 | 154 |
| 194 | 5 | 4 | 1 | 5 | 1 | 3 | 1 | 1 | 3 | 4 | 2 | 5 | 3 | 3 | 5 | 154 |
| 195 | 4 | 3 | 2 | 4 | 2 | 4 | 3 | 3 | 3 | 3 | 5 | 4 | 5 | 4 | 1 | 170 |
| 196 | 4 | 1 | 3 | 5 | 3 | 5 | 4 | 2 | 5 | 5 | 5 | 3 | 4 | 4 | 2 | 189 |
| 197 | 1 | 5 | 5 | 2 | 3 | 5 | 4 | 1 | 3 | 2 | 3 | 5 | 5 | 2 | 3 | 160 |
| 198 | 2 | 2 | 1 | 1 | 2 | 5 | 2 | 2 | 1 | 1 | 5 | 4 | 5 | 5 | 5 | 154 |
| 199 | 5 | 1 | 2 | 3 | 4 | 3 | 2 | 5 | 1 | 3 | 5 | 3 | 1 | 2 | 3 | 154 |
| 200 | 2 | 4 | 1 | 3 | 3 | 2 | 1 | 4 | 1 | 2 | 2 | 1 | 4 | 5 | 2 | 122 |
| 201 | 3 | 4 | 3 | 2 | 3 | 2 | 1 | 1 | 2 | 1 | 2 | 2 | 4 | 3 | 3 | 131 |
| 202 | 3 | 1 | 4 | 4 | 5 | 1 | 2 | 5 | 2 | 4 | 2 | 3 | 4 | 2 | 2 | 147 |
| 203 | 3 | 5 | 1 | 5 | 3 | 5 | 1 | 1 | 5 | 5 | 1 | 2 | 1 | 2 | 5 | 160 |
| 204 | 1 | 2 | 2 | 3 | 5 | 5 | 2 | 1 | 2 | 3 | 3 | 2 | 5 | 5 | 2 | 141 |
| 205 | 4 | 3 | 4 | 1 | 2 | 5 | 4 | 5 | 1 | 4 | 2 | 5 | 2 | 5 | 2 | 170 |
| 206 | 2 | 3 | 4 | 2 | 3 | 1 | 2 | 1 | 5 | 1 | 5 | 2 | 3 | 1 | 3 | 125 |
| 207 | 3 | 1 | 3 | 2 | 3 | 4 | 3 | 3 | 1 | 4 | 5 | 4 | 5 | 5 | 3 | 170 |
| 208 | 3 | 2 | 3 | 5 | 2 | 4 | 3 | 3 | 4 | 5 | 2 | 5 | 2 | 2 | 2 | 163 |
| 209 | 4 | 3 | 3 | 1 | 3 | 4 | 2 | 2 | 5 | 1 | 2 | 4 | 2 | 4 | 5 | 150 |
| 210 | 2 | 3 | 4 | 5 | 5 | 3 | 3 | 1 | 2 | 5 | 5 | 1 | 2 | 4 | 5 | 163 |
| 211 | 1 | 5 | 4 | 5 | 3 | 3 | 2 | 5 | 5 | 4 | 4 | 4 | 4 | 2 | 1 | 182 |
| 212 | 4 | 1 | 2 | 4 | 3 | 1 | 3 | 2 | 5 | 5 | 5 | 3 | 3 | 1 | 4 | 150 |
| 213 | 3 | 3 | 5 | 5 | 3 | 1 | 3 | 2 | 5 | 2 | 1 | 3 | 1 | 3 | 1 | 144 |
| 214 | 5 | 2 | 4 | 2 | 3 | 4 | 5 | 4 | 3 | 1 | 4 | 5 | 4 | 5 | 2 | 173 |
| 215 | 4 | 3 | 1 | 2 | 5 | 2 | 4 | 2 | 5 | 4 | 1 | 2 | 4 | 2 | 3 | 154 |
| 216 | 5 | 2 | 5 | 1 | 4 | 1 | 5 | 4 | 2 | 1 | 4 | 4 | 4 | 2 | 1 | 147 |
| 217 | 1 | 3 | 3 | 1 | 2 | 2 | 4 | 3 | 3 | 4 | 3 | 3 | 1 | 3 | 1 | 125 |
| 218 | 5 | 4 | 4 | 1 | 1 | 1 | 3 | 5 | 1 | 5 | 5 | 3 | 4 | 1 | 4 | 163 |
| 219 | 2 | 4 | 5 | 2 | 5 | 4 | 1 | 5 | 2 | 3 | 5 | 4 | 2 | 4 | 2 | 170 |
| 220 | 4 | 3 | 3 | 2 | 5 | 2 | 3 | 1 | 4 | 5 | 1 | 4 | 4 | 4 | 5 | 170 |
| 221 | 3 | 4 | 2 | 5 | 1 | 3 | 5 | 3 | 5 | 2 | 3 | 1 | 2 | 5 | 3 | 154 |
| 222 | 5 | 2 | 5 | 2 | 4 | 2 | 2 | 5 | 3 | 5 | 1 | 4 | 3 | 5 | 2 | 166 |
| 223 | 4 | 4 | 4 | 4 | 3 | 5 | 5 | 1 | 1 | 3 | 2 | 2 | 3 | 5 | 2 | 170 |
| 224 | 1 | 4 | 2 | 5 | 2 | 5 | 3 | 5 | 1 | 3 | 2 | 5 | 4 | 4 | 4 | 173 |
| 225 | 3 | 2 | 2 | 4 | 4 | 3 | 1 | 4 | 1 | 5 | 2 | 5 | 2 | 1 | 3 | 147 |
| 226 | 3 | 5 | 4 | 2 | 2 | 3 | 5 | 1 | 3 | 4 | 4 | 4 | 5 | 1 | 4 | 163 |
| 227 | 1 | 5 | 1 | 2 | 5 | 1 | 5 | 1 | 4 | 5 | 5 | 3 | 1 | 4 | 4 | 166 |
| 228 | 1 | 2 | 3 | 5 | 1 | 4 | 4 | 1 | 2 | 3 | 3 | 5 | 1 | 3 | 1 | 134 |
| 229 | 1 | 4 | 5 | 2 | 3 | 2 | 1 | 1 | 2 | 4 | 2 | 5 | 5 | 4 | 5 | 150 |
| 230 | 2 | 3 | 4 | 2 | 3 | 1 | 2 | 4 | 2 | 3 | 5 | 5 | 2 | 4 | 2 | 157 |
| 231 | 4 | 4 | 1 | 3 | 4 | 2 | 4 | 1 | 5 | 5 | 2 | 4 | 1 | 2 | 1 | 141 |
| 232 | 4 | 5 | 2 | 5 | 2 | 1 | 4 | 5 | 5 | 4 | 5 | 1 | 1 | 2 | 5 | 170 |
| 233 | 1 | 1 | 3 | 2 | 5 | 3 | 3 | 2 | 5 | 4 | 5 | 3 | 1 | 3 | 4 | 150 |
| 234 | 5 | 3 | 4 | 2 | 4 | 4 | 5 | 5 | 5 | 3 | 2 | 1 | 2 | 3 | 3 | 173 |
| 235 | 4 | 5 | 4 | 1 | 4 | 2 | 5 | 1 | 3 | 3 | 3 | 4 | 5 | 1 | 4 | 173 |
| 236 | 1 | 5 | 3 | 5 | 5 | 1 | 3 | 2 | 3 | 3 | 4 | 3 | 3 | 5 | 1 | 163 |
| 237 | 5 | 4 | 3 | 4 | 1 | 1 | 4 | 3 | 2 | 2 | 5 | 1 | 4 | 3 | 1 | 150 |
| 238 | 1 | 5 | 3 | 5 | 1 | 1 | 3 | 2 | 5 | 3 | 3 | 3 | 5 | 3 | 1 | 150 |
| 239 | 3 | 1 | 5 | 5 | 2 | 4 | 2 | 2 | 2 | 1 | 4 | 2 | 4 | 4 | 2 | 144 |
| 240 | 4 | 5 | 1 | 2 | 1 | 4 | 5 | 4 | 1 | 5 | 3 | 1 | 1 | 2 | 1 | 138 |
| 241 | 1 | 4 | 4 | 1 | 4 | 1 | 1 | 4 | 3 | 1 | 4 | 5 | 2 | 2 | 1 | 134 |
| 242 | 3 | 1 | 1 | 1 | 3 | 5 | 5 | 1 | 2 | 5 | 5 | 4 | 2 | 1 | 1 | 141 |
| 243 | 1 | 3 | 3 | 1 | 4 | 3 | 2 | 4 | 3 | 5 | 4 | 2 | 1 | 2 | 1 | 141 |
| 244 | 1 | 3 | 4 | 5 | 1 | 2 | 5 | 5 | 1 | 1 | 3 | 2 | 1 | 4 | 5 | 147 |
| 245 | 2 | 5 | 2 | 3 | 4 | 1 | 5 | 2 | 4 | 1 | 4 | 3 | 1 | 2 | 5 | 150 |
| 246 | 4 | 1 | 2 | 4 | 4 | 5 | 1 | 1 | 5 | 3 | 3 | 4 | 4 | 2 | 4 | 160 |
| 247 | 1 | 1 | 1 | 4 | 2 | 1 | 5 | 4 | 2 | 1 | 5 | 3 | 1 | 3 | 5 | 131 |
| 248 | 4 | 2 | 4 | 3 | 2 | 5 | 4 | 2 | 4 | 4 | 5 | 4 | 3 | 3 | 1 | 170 |
| 249 | 4 | 4 | 2 | 3 | 4 | 2 | 1 | 2 | 5 | 4 | 1 | 2 | 5 | 5 | 4 | 166 |
| 250 | 5 | 2 | 5 | 5 | 3 | 1 | 2 | 4 | 3 | 1 | 2 | 2 | 2 | 4 | 4 | 150 |
| 251 | 4 | 5 | 1 | 4 | 1 | 5 | 5 | 1 | 3 | 5 | 2 | 2 | 2 | 2 | 3 | 157 |
| 252 | 3 | 2 | 4 | 5 | 2 | 4 | 1 | 3 | 5 | 1 | 3 | 1 | 1 | 5 | 4 | 150 |
| 253 | 3 | 4 | 4 | 2 | 1 | 5 | 3 | 5 | 5 | 4 | 2 | 5 | 5 | 5 | 4 | 192 |
| 254 | 2 | 4 | 3 | 4 | 4 | 1 | 4 | 4 | 5 | 2 | 3 | 5 | 2 | 5 | 2 | 166 |
| 255 | 3 | 4 | 1 | 3 | 3 | 1 | 5 | 2 | 3 | 2 | 1 | 3 | 3 | 1 | 3 | 125 |
| 256 | 2 | 4 | 3 | 3 | 5 | 2 | 5 | 5 | 1 | 5 | 1 | 1 | 3 | 2 | 1 | 144 |
| 257 | 4 | 1 | 2 | 4 | 1 | 3 | 2 | 1 | 2 | 1 | 2 | 1 | 1 | 4 | 5 | 118 |
| 258 | 1 | 3 | 4 | 3 | 2 | 3 | 5 | 1 | 2 | 4 | 3 | 3 | 5 | 3 | 4 | 154 |
| 259 | 2 | 1 | 4 | 1 | 2 | 4 | 1 | 5 | 2 | 2 | 5 | 3 | 1 | 5 | 5 | 141 |
| 260 | 5 | 1 | 5 | 1 | 5 | 3 | 2 | 5 | 3 | 2 | 4 | 1 | 4 | 4 | 2 | 154 |
| 261 | 3 | 3 | 1 | 3 | 4 | 2 | 2 | 5 | 2 | 5 | 5 | 2 | 3 | 3 | 2 | 150 |
| 262 | 3 | 2 | 2 | 2 | 3 | 3 | 1 | 1 | 1 | 1 | 3 | 5 | 4 | 3 | 1 | 122 |
| 263 | 3 | 3 | 4 | 5 | 2 | 1 | 4 | 2 | 1 | 4 | 4 | 2 | 4 | 5 | 1 | 150 |
| 264 | 2 | 2 | 5 | 2 | 1 | 1 | 2 | 2 | 2 | 1 | 5 | 2 | 1 | 1 | 3 | 112 |
| 265 | 4 | 2 | 3 | 1 | 3 | 2 | 1 | 3 | 3 | 1 | 1 | 3 | 2 | 3 | 1 | 109 |
| 266 | 2 | 3 | 3 | 3 | 1 | 3 | 1 | 3 | 4 | 2 | 5 | 2 | 3 | 4 | 2 | 138 |
| 267 | 1 | 4 | 1 | 4 | 3 | 3 | 4 | 5 | 5 | 5 | 2 | 1 | 3 | 1 | 4 | 154 |
| 268 | 1 | 5 | 2 | 4 | 3 | 4 | 5 | 3 | 1 | 1 | 4 | 1 | 1 | 3 | 1 | 128 |
| 269 | 5 | 2 | 3 | 3 | 1 | 1 | 2 | 4 | 3 | 5 | 2 | 3 | 2 | 3 | 3 | 147 |
| 270 | 5 | 3 | 3 | 1 | 2 | 5 | 4 | 4 | 1 | 2 | 2 | 3 | 2 | 1 | 3 | 138 |
| 271 | 5 | 5 | 2 | 5 | 2 | 3 | 2 | 3 | 5 | 1 | 5 | 2 | 2 | 4 | 4 | 176 |
| 272 | 4 | 2 | 3 | 1 | 3 | 4 | 5 | 1 | 3 | 2 | 5 | 4 | 1 | 5 | 2 | 150 |
| 273 | 4 | 5 | 2 | 2 | 1 | 3 | 3 | 1 | 4 | 1 | 5 | 5 | 4 | 5 | 1 | 160 |
| 274 | 3 | 4 | 5 | 1 | 4 | 4 | 1 | 3 | 4 | 1 | 4 | 3 | 5 | 5 | 4 | 170 |
| 275 | 2 | 4 | 5 | 2 | 5 | 1 | 1 | 4 | 1 | 2 | 5 | 4 | 3 | 1 | 4 | 147 |
| 276 | 5 | 1 | 4 | 4 | 4 | 1 | 5 | 5 | 4 | 4 | 4 | 4 | 4 | 2 | 2 | 176 |
| 277 | 2 | 4 | 4 | 1 | 3 | 3 | 4 | 3 | 3 | 5 | 5 | 1 | 1 | 1 | 1 | 134 |
| 278 | 2 | 2 | 2 | 1 | 2 | 3 | 1 | 4 | 4 | 2 | 4 | 1 | 4 | 2 | 4 | 131 |
| 279 | 1 | 1 | 5 | 4 | 4 | 1 | 1 | 2 | 2 | 5 | 4 | 4 | 5 | 5 | 4 | 160 |
| 280 | 5 | 2 | 5 | 5 | 4 | 5 | 3 | 5 | 5 | 3 | 3 | 3 | 4 | 5 | 1 | 189 |
| 281 | 3 | 1 | 5 | 4 | 4 | 5 | 3 | 4 | 4 | 4 | 2 | 1 | 4 | 5 | 4 | 179 |
| 282 | 3 | 1 | 2 | 4 | 4 | 4 | 3 | 4 | 3 | 4 | 5 | 5 | 5 | 3 | 3 | 173 |
| 283 | 2 | 2 | 3 | 5 | 3 | 5 | 1 | 2 | 5 | 3 | 3 | 4 | 5 | 4 | 1 | 166 |
| 284 | 1 | 2 | 2 | 4 | 1 | 3 | 3 | 2 | 1 | 5 | 4 | 3 | 4 | 3 | 5 | 144 |
| 285 | 2 | 5 | 1 | 4 | 3 | 3 | 5 | 3 | 1 | 4 | 1 | 2 | 3 | 2 | 4 | 147 |
| 286 | 5 | 3 | 5 | 1 | 5 | 2 | 3 | 1 | 1 | 5 | 5 | 5 | 2 | 1 | 4 | 170 |
| 287 | 1 | 2 | 1 | 1 | 4 | 1 | 4 | 4 | 4 | 5 | 5 | 5 | 1 | 4 | 2 | 157 |
| 288 | 5 | 4 | 4 | 4 | 1 | 4 | 4 | 5 | 4 | 2 | 4 | 5 | 5 | 5 | 1 | 186 |
| 289 | 1 | 3 | 1 | 1 | 1 | 1 | 3 | 2 | 3 | 2 | 4 | 3 | 5 | 1 | 5 | 122 |
| 290 | 2 | 4 | 5 | 4 | 2 | 5 | 1 | 3 | 4 | 2 | 2 | 1 | 4 | 1 | 3 | 141 |
| 291 | 1 | 3 | 1 | 1 | 3 | 3 | 3 | 5 | 5 | 4 | 3 | 1 | 3 | 2 | 2 | 138 |
| 292 | 2 | 4 | 5 | 1 | 5 | 2 | 4 | 1 | 4 | 3 | 5 | 4 | 4 | 4 | 5 | 182 |
| 293 | 1 | 3 | 5 | 3 | 2 | 2 | 3 | 1 | 4 | 5 | 4 | 4 | 2 | 5 | 5 | 173 |
| 294 | 3 | 5 | 4 | 4 | 5 | 1 | 2 | 2 | 2 | 1 | 4 | 1 | 2 | 2 | 5 | 141 |
| 295 | 1 | 2 | 2 | 3 | 1 | 3 | 4 | 3 | 4 | 5 | 3 | 5 | 4 | 4 | 2 | 160 |
| 296 | 5 | 4 | 5 | 4 | 2 | 1 | 1 | 5 | 5 | 1 | 5 | 3 | 1 | 3 | 3 | 166 |
| 297 | 3 | 3 | 2 | 1 | 1 | 5 | 1 | 1 | 4 | 5 | 5 | 2 | 4 | 2 | 3 | 141 |
| 298 | 3 | 4 | 5 | 5 | 3 | 4 | 4 | 2 | 3 | 4 | 2 | 1 | 2 | 5 | 3 | 163 |
| 299 | 4 | 2 | 5 | 2 | 4 | 1 | 2 | 4 | 4 | 3 | 1 | 4 | 5 | 1 | 5 | 166 |
| 300 | 1 | 2 | 2 | 5 | 3 | 5 | 4 | 1 | 3 | 4 | 1 | 2 | 2 | 4 | 3 | 138 |
| 301 | 2 | 4 | 3 | 5 | 3 | 3 | 3 | 5 | 3 | 1 | 3 | 5 | 3 | 2 | 4 | 170 |
| 302 | 1 | 3 | 2 | 2 | 2 | 4 | 5 | 3 | 1 | 1 | 3 | 3 | 1 | 5 | 3 | 141 |
| 303 | 5 | 3 | 2 | 2 | 5 | 2 | 1 | 5 | 3 | 3 | 2 | 4 | 5 | 3 | 3 | 160 |
| 304 | 3 | 1 | 5 | 4 | 1 | 2 | 1 | 3 | 5 | 4 | 3 | 2 | 3 | 2 | 1 | 134 |
| 305 | 3 | 2 | 5 | 2 | 1 | 5 | 3 | 1 | 4 | 1 | 5 | 4 | 4 | 2 | 1 | 141 |
| 306 | 2 | 3 | 2 | 5 | 3 | 5 | 4 | 1 | 5 | 2 | 1 | 5 | 1 | 5 | 4 | 163 |
| 307 | 4 | 5 | 4 | 1 | 3 | 1 | 2 | 3 | 2 | 2 | 3 | 5 | 4 | 5 | 2 | 157 |
| 308 | 3 | 1 | 3 | 5 | 2 | 1 | 4 | 5 | 3 | 2 | 5 | 1 | 1 | 2 | 3 | 134 |
| 309 | 3 | 3 | 3 | 5 | 3 | 4 | 4 | 1 | 4 | 4 | 5 | 4 | 5 | 2 | 1 | 170 |
| 310 | 1 | 2 | 2 | 5 | 1 | 1 | 2 | 2 | 3 | 2 | 1 | 4 | 4 | 5 | 1 | 122 |
| 311 | 1 | 4 | 3 | 2 | 1 | 5 | 2 | 1 | 5 | 2 | 2 | 5 | 4 | 4 | 4 | 154 |
| 312 | 5 | 5 | 5 | 4 | 2 | 1 | 4 | 4 | 5 | 5 | 2 | 4 | 5 | 3 | 5 | 195 |
| 313 | 4 | 5 | 3 | 4 | 4 | 4 | 3 | 1 | 3 | 1 | 1 | 5 | 1 | 2 | 4 | 160 |
| 314 | 4 | 1 | 5 | 1 | 2 | 5 | 1 | 2 | 2 | 5 | 2 | 3 | 5 | 2 | 1 | 144 |
| 315 | 2 | 4 | 3 | 3 | 2 | 3 | 3 | 1 | 2 | 4 | 1 | 1 | 4 | 1 | 4 | 128 |
| 316 | 3 | 2 | 4 | 5 | 4 | 2 | 3 | 3 | 2 | 2 | 4 | 5 | 5 | 4 | 3 | 166 |
| 317 | 4 | 4 | 2 | 2 | 4 | 5 | 5 | 5 | 3 | 1 | 4 | 1 | 2 | 3 | 5 | 173 |
| 318 | 2 | 3 | 4 | 2 | 4 | 1 | 5 | 4 | 3 | 4 | 5 | 2 | 4 | 4 | 2 | 170 |
| 319 | 3 | 2 | 4 | 5 | 2 | 5 | 1 | 4 | 1 | 2 | 1 | 4 | 5 | 2 | 2 | 144 |
| 320 | 4 | 4 | 5 | 3 | 3 | 2 | 1 | 2 | 4 | 5 | 2 | 5 | 4 | 1 | 1 | 157 |
| 321 | 1 | 5 | 1 | 2 | 2 | 3 | 3 | 5 | 4 | 2 | 4 | 3 | 1 | 1 | 4 | 134 |
| 322 | 3 | 5 | 4 | 4 | 3 | 4 | 4 | 1 | 3 | 5 | 1 | 1 | 2 | 4 | 2 | 154 |
| 323 | 1 | 4 | 4 | 3 | 3 | 5 | 5 | 1 | 2 | 1 | 2 | 2 | 3 | 2 | 1 | 138 |
| 324 | 3 | 4 | 2 | 5 | 2 | 2 | 5 | 5 | 4 | 4 | 4 | 5 | 3 | 3 | 3 | 176 |
| 325 | 3 | 5 | 2 | 2 | 4 | 1 | 1 | 5 | 4 | 2 | 3 | 1 | 5 | 4 | 3 | 150 |
| 326 | 2 | 3 | 2 | 4 | 5 | 5 | 4 | 3 | 2 | 5 | 5 | 1 | 2 | 5 | 5 | 173 |
| 327 | 3 | 3 | 4 | 5 | 3 | 5 | 4 | 5 | 4 | 2 | 1 | 1 | 5 | 4 | 3 | 179 |
| 328 | 4 | 2 | 4 | 1 | 3 | 1 | 5 | 2 | 2 | 2 | 4 | 3 | 2 | 3 | 3 | 144 |
| 329 | 2 | 4 | 1 | 4 | 4 | 3 | 2 | 4 | 2 | 1 | 4 | 2 | 1 | 5 | 4 | 150 |
| 330 | 1 | 2 | 5 | 2 | 3 | 5 | 5 | 3 | 3 | 2 | 3 | 2 | 5 | 1 | 4 | 154 |
| 331 | 1 | 5 | 1 | 1 | 1 | 5 | 4 | 4 | 4 | 5 | 4 | 1 | 5 | 2 | 3 | 160 |
| 332 | 4 | 5 | 1 | 5 | 5 | 4 | 1 | 4 | 3 | 4 | 2 | 2 | 3 | 4 | 1 | 157 |
| 333 | 1 | 1 | 2 | 5 | 1 | 2 | 1 | 2 | 3 | 5 | 3 | 1 | 3 | 2 | 1 | 122 |
| 334 | 1 | 3 | 3 | 4 | 5 | 3 | 2 | 5 | 3 | 5 | 3 | 5 | 2 | 1 | 3 | 170 |
| 335 | 1 | 5 | 4 | 3 | 2 | 3 | 3 | 2 | 4 | 4 | 5 | 5 | 1 | 1 | 2 | 160 |
| 336 | 4 | 2 | 2 | 1 | 1 | 4 | 3 | 1 | 3 | 2 | 4 | 4 | 2 | 2 | 3 | 138 |
| 337 | 2 | 5 | 2 | 3 | 3 | 5 | 3 | 4 | 3 | 3 | 5 | 3 | 3 | 4 | 5 | 176 |
| 338 | 1 | 1 | 3 | 4 | 4 | 4 | 5 | 3 | 3 | 5 | 5 | 1 | 4 | 1 | 2 | 160 |
| 339 | 4 | 4 | 4 | 4 | 3 | 4 | 1 | 5 | 5 | 3 | 1 | 5 | 2 | 4 | 1 | 166 |
| 340 | 4 | 1 | 5 | 3 | 5 | 4 | 2 | 3 | 2 | 2 | 3 | 2 | 2 | 4 | 4 | 163 |
| 341 | 2 | 5 | 2 | 3 | 1 | 1 | 1 | 1 | 5 | 4 | 5 | 4 | 1 | 4 | 4 | 154 |
| 342 | 1 | 1 | 1 | 1 | 4 | 3 | 4 | 4 | 4 | 1 | 1 | 2 | 5 | 2 | 3 | 134 |
| 343 | 4 | 5 | 2 | 5 | 2 | 5 | 3 | 1 | 5 | 2 | 1 | 4 | 3 | 5 | 1 | 160 |
| 344 | 5 | 5 | 4 | 4 | 1 | 2 | 2 | 4 | 3 | 5 | 2 | 3 | 1 | 1 | 3 | 154 |
| 345 | 3 | 3 | 2 | 5 | 1 | 4 | 3 | 2 | 1 | 1 | 4 | 1 | 1 | 5 | 4 | 131 |
| 346 | 3 | 4 | 2 | 2 | 3 | 1 | 4 | 2 | 5 | 4 | 3 | 1 | 2 | 4 | 3 | 141 |
| 347 | 3 | 5 | 4 | 5 | 5 | 4 | 3 | 5 | 5 | 2 | 1 | 2 | 4 | 3 | 1 | 170 |
| 348 | 1 | 1 | 5 | 2 | 4 | 4 | 5 | 4 | 2 | 4 | 2 | 4 | 3 | 5 | 2 | 166 |
| 349 | 4 | 5 | 4 | 3 | 5 | 1 | 5 | 5 | 5 | 5 | 5 | 5 | 1 | 3 | 1 | 198 |
| 350 | 4 | 4 | 3 | 1 | 4 | 1 | 4 | 5 | 4 | 4 | 2 | 3 | 3 | 1 | 5 | 163 |
| 351 | 4 | 5 | 1 | 4 | 5 | 3 | 5 | 3 | 1 | 3 | 5 | 2 | 2 | 2 | 3 | 160 |
| 352 | 2 | 4 | 2 | 5 | 5 | 4 | 4 | 4 | 5 | 3 | 2 | 1 | 2 | 3 | 5 | 173 |
| 353 | 4 | 2 | 5 | 5 | 2 | 1 | 1 | 1 | 2 | 2 | 3 | 5 | 1 | 4 | 4 | 141 |
| 354 | 3 | 2 | 5 | 5 | 5 | 4 | 3 | 2 | 2 | 5 | 3 | 5 | 5 | 4 | 1 | 186 |
| 355 | 4 | 2 | 3 | 4 | 1 | 5 | 5 | 1 | 2 | 3 | 2 | 4 | 2 | 5 | 4 | 154 |
| 356 | 3 | 2 | 4 | 5 | 1 | 3 | 2 | 5 | 4 | 3 | 3 | 3 | 3 | 2 | 3 | 157 |
| 357 | 2 | 3 | 4 | 5 | 5 | 3 | 4 | 3 | 2 | 3 | 2 | 2 | 1 | 3 | 4 | 157 |
| 358 | 1 | 2 | 4 | 1 | 2 | 1 | 5 | 4 | 3 | 4 | 4 | 2 | 3 | 3 | 4 | 150 |
| 359 | 2 | 3 | 5 | 3 | 1 | 1 | 2 | 3 | 2 | 2 | 5 | 1 | 1 | 4 | 4 | 128 |
| 360 | 2 | 1 | 2 | 2 | 2 | 4 | 1 | 3 | 2 | 3 | 2 | 5 | 4 | 1 | 5 | 138 |
| 361 | 3 | 2 | 3 | 2 | 2 | 2 | 1 | 3 | 1 | 3 | 1 | 5 | 4 | 3 | 1 | 118 |
| 362 | 2 | 3 | 5 | 2 | 2 | 1 | 2 | 2 | 1 | 3 | 1 | 1 | 2 | 5 | 5 | 134 |
| 363 | 4 | 1 | 4 | 1 | 2 | 5 | 5 | 4 | 5 | 1 | 5 | 5 | 5 | 4 | 3 | 189 |
| 364 | 2 | 5 | 4 | 2 | 1 | 2 | 1 | 1 | 4 | 4 | 4 | 3 | 4 | 5 | 2 | 150 |
| 365 | 3 | 4 | 3 | 3 | 2 | 3 | 2 | 5 | 4 | 1 | 1 | 2 | 4 | 3 | 1 | 141 |
| 366 | 2 | 2 | 5 | 1 | 1 | 4 | 5 | 3 | 5 | 4 | 3 | 1 | 1 | 3 | 5 | 160 |
| 367 | 3 | 3 | 4 | 1 | 5 | 4 | 3 | 2 | 2 | 4 | 3 | 2 | 3 | 5 | 2 | 150 |
| 368 | 3 | 1 | 5 | 4 | 4 | 1 | 1 | 3 | 1 | 3 | 3 | 1 | 1 | 3 | 5 | 131 |
| 369 | 4 | 4 | 1 | 4 | 1 | 2 | 1 | 2 | 5 | 4 | 1 | 1 | 4 | 2 | 2 | 125 |
| 370 | 2 | 5 | 1 | 3 | 3 | 4 | 2 | 2 | 3 | 2 | 5 | 1 | 5 | 3 | 5 | 150 |
| 371 | 2 | 1 | 3 | 2 | 1 | 3 | 2 | 4 | 2 | 1 | 3 | 5 | 5 | 5 | 2 | 141 |
| 372 | 2 | 3 | 1 | 2 | 3 | 3 | 2 | 5 | 2 | 1 | 3 | 2 | 1 | 2 | 1 | 115 |
| 373 | 3 | 1 | 4 | 4 | 5 | 5 | 3 | 5 | 1 | 3 | 5 | 1 | 2 | 5 | 5 | 170 |
| 374 | 1 | 5 | 3 | 3 | 5 | 5 | 5 | 5 | 1 | 1 | 4 | 3 | 5 | 4 | 2 | 179 |
| 375 | 2 | 2 | 1 | 1 | 2 | 3 | 3 | 5 | 2 | 2 | 2 | 3 | 5 | 3 | 4 | 144 |
| 376 | 5 | 5 | 3 | 2 | 4 | 5 | 4 | 1 | 4 | 5 | 2 | 1 | 2 | 2 | 3 | 163 |
| 377 | 1 | 5 | 3 | 1 | 4 | 4 | 5 | 1 | 3 | 1 | 5 | 1 | 4 | 1 | 1 | 144 |
| 378 | 2 | 4 | 2 | 2 | 1 | 4 | 4 | 3 | 2 | 3 | 1 | 5 | 2 | 2 | 2 | 131 |
| 379 | 1 | 5 | 5 | 4 | 5 | 3 | 3 | 4 | 4 | 4 | 5 | 4 | 5 | 1 | 1 | 176 |
| 380 | 5 | 4 | 4 | 2 | 5 | 4 | 4 | 3 | 4 | 1 | 5 | 1 | 4 | 3 | 2 | 179 |
| 381 | 2 | 4 | 1 | 1 | 4 | 3 | 1 | 2 | 1 | 1 | 1 | 2 | 5 | 5 | 1 | 115 |
| 382 | 2 | 5 | 5 | 3 | 3 | 1 | 2 | 5 | 4 | 2 | 1 | 1 | 2 | 5 | 5 | 150 |
| 383 | 4 | 1 | 1 | 2 | 5 | 1 | 3 | 3 | 2 | 3 | 1 | 2 | 3 | 1 | 4 | 125 |
| 384 | 2 | 5 | 5 | 4 | 5 | 3 | 4 | 5 | 4 | 4 | 4 | 1 | 5 | 4 | 4 | 205 |
| 385 | 4 | 4 | 1 | 4 | 2 | 5 | 4 | 2 | 3 | 5 | 4 | 3 | 3 | 2 | 5 | 166 |
| 386 | 5 | 3 | 4 | 4 | 4 | 5 | 3 | 1 | 4 | 4 | 2 | 2 | 4 | 3 | 1 | 173 |
| 387 | 4 | 4 | 1 | 5 | 3 | 5 | 4 | 2 | 3 | 3 | 5 | 3 | 4 | 2 | 1 | 163 |
| 388 | 1 | 3 | 1 | 4 | 3 | 2 | 1 | 2 | 1 | 5 | 5 | 2 | 2 | 5 | 3 | 144 |
| 389 | 2 | 5 | 1 | 1 | 5 | 2 | 4 | 4 | 5 | 2 | 5 | 3 | 2 | 5 | 1 | 166 |
| 390 | 4 | 3 | 3 | 5 | 4 | 5 | 4 | 1 | 3 | 3 | 2 | 5 | 5 | 3 | 2 | 182 |
| 391 | 4 | 5 | 3 | 2 | 2 | 4 | 1 | 1 | 3 | 2 | 3 | 2 | 4 | 1 | 2 | 131 |
| 392 | 3 | 3 | 4 | 1 | 1 | 4 | 5 | 1 | 2 | 2 | 4 | 4 | 5 | 2 | 3 | 150 |
| 393 | 5 | 3 | 1 | 5 | 4 | 2 | 5 | 1 | 3 | 3 | 5 | 4 | 4 | 1 | 4 | 166 |
| 394 | 4 | 5 | 5 | 1 | 3 | 2 | 3 | 5 | 1 | 5 | 2 | 3 | 2 | 2 | 5 | 166 |
| 395 | 4 | 2 | 1 | 1 | 3 | 4 | 2 | 3 | 4 | 4 | 4 | 2 | 2 | 5 | 5 | 154 |
| 396 | 5 | 4 | 2 | 2 | 2 | 1 | 1 | 4 | 3 | 5 | 1 | 3 | 3 | 2 | 2 | 134 |
| 397 | 4 | 2 | 4 | 1 | 2 | 5 | 4 | 4 | 2 | 3 | 1 | 4 | 3 | 2 | 1 | 138 |
| 398 | 4 | 3 | 1 | 2 | 4 | 4 | 2 | 3 | 5 | 5 | 4 | 2 | 5 | 3 | 5 | 170 |
| 399 | 1 | 3 | 1 | 5 | 3 | 2 | 2 | 3 | 2 | 3 | 2 | 5 | 5 | 4 | 5 | 157 |
| 400 | 2 | 1 | 1 | 5 | 4 | 2 | 3 | 2 | 4 | 1 | 3 | 4 | 2 | 3 | 2 | 128 |
| 401 | 4 | 3 | 3 | 4 | 1 | 4 | 2 | 1 | 2 | 4 | 4 | 2 | 2 | 5 | 2 | 144 |
| 402 | 2 | 5 | 3 | 5 | 1 | 1 | 5 | 4 | 2 | 2 | 5 | 3 | 5 | 1 | 4 | 166 |
| 403 | 3 | 4 | 4 | 3 | 2 | 2 | 3 | 1 | 4 | 3 | 2 | 4 | 1 | 3 | 1 | 138 |
| 404 | 5 | 3 | 4 | 2 | 1 | 2 | 2 | 3 | 3 | 3 | 1 | 2 | 2 | 3 | 2 | 128 |
| 405 | 5 | 1 | 5 | 2 | 2 | 2 | 1 | 5 | 4 | 3 | 3 | 3 | 5 | 5 | 1 | 154 |
| 406 | 2 | 5 | 1 | 4 | 5 | 1 | 1 | 2 | 3 | 3 | 5 | 4 | 1 | 1 | 3 | 144 |
| 407 | 1 | 2 | 5 | 5 | 1 | 3 | 1 | 3 | 3 | 1 | 2 | 1 | 1 | 1 | 2 | 109 |
| 408 | 3 | 4 | 3 | 1 | 2 | 3 | 3 | 5 | 2 | 3 | 2 | 1 | 4 | 4 | 1 | 141 |
| 409 | 5 | 5 | 4 | 5 | 3 | 5 | 4 | 4 | 3 | 4 | 2 | 2 | 4 | 3 | 3 | 186 |
| 410 | 4 | 1 | 2 | 3 | 4 | 2 | 4 | 3 | 4 | 3 | 3 | 3 | 1 | 2 | 5 | 150 |
| 411 | 5 | 1 | 1 | 5 | 1 | 2 | 3 | 2 | 5 | 1 | 2 | 5 | 4 | 5 | 3 | 147 |
| 412 | 1 | 5 | 3 | 5 | 3 | 5 | 1 | 4 | 4 | 5 | 5 | 1 | 5 | 2 | 3 | 176 |
| 413 | 5 | 5 | 2 | 1 | 5 | 3 | 2 | 3 | 2 | 3 | 2 | 3 | 1 | 4 | 3 | 147 |
| 414 | 1 | 2 | 3 | 1 | 5 | 4 | 1 | 2 | 4 | 4 | 1 | 4 | 5 | 5 | 4 | 157 |
| 415 | 3 | 4 | 4 | 3 | 2 | 4 | 1 | 4 | 2 | 4 | 3 | 5 | 4 | 4 | 3 | 163 |
| 416 | 4 | 4 | 2 | 1 | 2 | 5 | 5 | 1 | 1 | 5 | 3 | 2 | 2 | 2 | 2 | 144 |
| 417 | 3 | 1 | 3 | 4 | 1 | 4 | 2 | 3 | 5 | 2 | 1 | 2 | 2 | 5 | 5 | 144 |
| 418 | 1 | 1 | 1 | 5 | 1 | 5 | 5 | 3 | 2 | 5 | 4 | 1 | 3 | 5 | 2 | 150 |
| 419 | 3 | 2 | 1 | 4 | 5 | 4 | 3 | 5 | 5 | 3 | 3 | 2 | 5 | 2 | 2 | 173 |
| 420 | 2 | 4 | 4 | 2 | 4 | 1 | 1 | 1 | 2 | 1 | 3 | 5 | 3 | 2 | 1 | 131 |
| 421 | 5 | 2 | 5 | 4 | 4 | 2 | 1 | 1 | 3 | 4 | 2 | 5 | 4 | 2 | 5 | 173 |
| 422 | 2 | 4 | 3 | 3 | 1 | 5 | 2 | 2 | 5 | 3 | 4 | 1 | 5 | 3 | 5 | 166 |
| 423 | 1 | 2 | 5 | 3 | 4 | 2 | 5 | 3 | 5 | 4 | 1 | 1 | 4 | 1 | 5 | 150 |
| 424 | 5 | 2 | 2 | 5 | 2 | 2 | 4 | 2 | 2 | 1 | 4 | 5 | 3 | 4 | 3 | 150 |
| 425 | 1 | 2 | 4 | 4 | 3 | 4 | 4 | 5 | 3 | 4 | 1 | 2 | 5 | 4 | 1 | 157 |
| 426 | 3 | 4 | 5 | 5 | 4 | 2 | 3 | 3 | 5 | 3 | 4 | 2 | 4 | 4 | 1 | 179 |
| 427 | 1 | 2 | 4 | 5 | 3 | 5 | 3 | 3 | 4 | 2 | 1 | 2 | 1 | 1 | 1 | 128 |
| 428 | 2 | 2 | 1 | 2 | 5 | 2 | 4 | 2 | 3 | 3 | 1 | 2 | 3 | 4 | 1 | 125 |
| 429 | 1 | 1 | 1 | 5 | 1 | 1 | 4 | 2 | 2 | 2 | 3 | 5 | 3 | 4 | 3 | 131 |
| 430 | 3 | 3 | 1 | 5 | 3 | 1 | 5 | 2 | 3 | 5 | 3 | 1 | 5 | 2 | 1 | 144 |
| 431 | 4 | 2 | 2 | 4 | 1 | 2 | 4 | 1 | 5 | 3 | 1 | 4 | 3 | 1 | 4 | 138 |
| 432 | 3 | 3 | 3 | 3 | 2 | 3 | 3 | 2 | 1 | 1 | 1 | 5 | 2 | 5 | 4 | 141 |
| 433 | 4 | 2 | 2 | 4 | 2 | 2 | 3 | 5 | 2 | 5 | 1 | 3 | 1 | 5 | 4 | 160 |
| 434 | 2 | 5 | 1 | 2 | 4 | 4 | 1 | 5 | 3 | 4 | 1 | 1 | 4 | 1 | 3 | 144 |
| 435 | 2 | 1 | 2 | 1 | 1 | 2 | 3 | 5 | 4 | 1 | 5 | 3 | 5 | 1 | 4 | 134 |
| 436 | 1 | 2 | 5 | 4 | 1 | 2 | 5 | 4 | 5 | 1 | 4 | 2 | 4 | 5 | 5 | 173 |
| 437 | 4 | 5 | 3 | 4 | 1 | 5 | 3 | 3 | 4 | 4 | 4 | 2 | 3 | 1 | 1 | 157 |
| 438 | 5 | 4 | 1 | 1 | 3 | 1 | 3 | 1 | 2 | 5 | 1 | 3 | 3 | 3 | 2 | 131 |
| 439 | 1 | 5 | 3 | 3 | 4 | 4 | 3 | 2 | 4 | 5 | 4 | 3 | 5 | 1 | 3 | 173 |
| 440 | 2 | 2 | 3 | 5 | 1 | 2 | 2 | 3 | 3 | 5 | 2 | 5 | 3 | 3 | 3 | 147 |
| 441 | 3 | 1 | 5 | 3 | 4 | 1 | 3 | 4 | 1 | 1 | 4 | 3 | 4 | 4 | 1 | 138 |
| 442 | 4 | 4 | 4 | 4 | 2 | 5 | 3 | 4 | 5 | 5 | 5 | 3 | 4 | 3 | 2 | 198 |
| 443 | 1 | 2 | 5 | 1 | 3 | 1 | 2 | 2 | 5 | 1 | 1 | 4 | 5 | 1 | 3 | 128 |
| 444 | 1 | 1 | 1 | 2 | 4 | 5 | 4 | 3 | 5 | 3 | 5 | 4 | 4 | 4 | 5 | 166 |
| 445 | 4 | 3 | 2 | 2 | 3 | 4 | 4 | 5 | 2 | 5 | 4 | 5 | 4 | 1 | 5 | 186 |
| 446 | 5 | 1 | 4 | 2 | 1 | 1 | 2 | 1 | 4 | 5 | 3 | 1 | 3 | 5 | 2 | 144 |
| 447 | 3 | 3 | 2 | 2 | 1 | 1 | 5 | 1 | 4 | 5 | 3 | 4 | 2 | 3 | 5 | 150 |
| 448 | 1 | 5 | 2 | 4 | 1 | 5 | 2 | 3 | 5 | 4 | 2 | 3 | 5 | 5 | 1 | 170 |
| 449 | 1 | 1 | 1 | 2 | 3 | 4 | 1 | 5 | 3 | 3 | 4 | 3 | 4 | 3 | 5 | 150 |
| 450 | 1 | 3 | 5 | 2 | 2 | 3 | 4 | 5 | 5 | 3 | 2 | 5 | 3 | 3 | 1 | 166 |
| 451 | 1 | 2 | 3 | 2 | 2 | 1 | 1 | 1 | 1 | 3 | 1 | 4 | 3 | 3 | 1 | 109 |
| 452 | 1 | 5 | 5 | 2 | 3 | 5 | 3 | 4 | 3 | 1 | 1 | 1 | 2 | 4 | 5 | 157 |
| 453 | 2 | 2 | 2 | 1 | 4 | 3 | 2 | 5 | 1 | 3 | 2 | 4 | 1 | 1 | 4 | 134 |
| 454 | 5 | 2 | 5 | 3 | 1 | 1 | 2 | 5 | 1 | 5 | 4 | 1 | 3 | 4 | 1 | 147 |
| 455 | 5 | 1 | 3 | 4 | 1 | 2 | 3 | 3 | 4 | 3 | 5 | 1 | 2 | 2 | 2 | 147 |
| 456 | 5 | 1 | 4 | 1 | 5 | 5 | 5 | 4 | 4 | 5 | 4 | 3 | 4 | 2 | 4 | 186 |
| 457 | 1 | 2 | 1 | 1 | 2 | 2 | 1 | 1 | 5 | 3 | 2 | 4 | 3 | 5 | 1 | 122 |
| 458 | 4 | 1 | 4 | 1 | 5 | 5 | 1 | 2 | 2 | 3 | 5 | 1 | 3 | 2 | 4 | 147 |
| 459 | 1 | 4 | 1 | 2 | 5 | 2 | 3 | 4 | 1 | 2 | 5 | 5 | 5 | 5 | 5 | 176 |
| 460 | 5 | 2 | 1 | 5 | 5 | 5 | 1 | 5 | 1 | 4 | 3 | 3 | 2 | 4 | 3 | 170 |
| 461 | 5 | 1 | 3 | 4 | 4 | 5 | 1 | 2 | 3 | 1 | 1 | 4 | 5 | 4 | 5 | 157 |
